# Cyclopentene ring effects in cyanine dyes: a handle to fine-tune photophysical properties†

**DOI:** 10.1039/d3cp05219b

**Published:** 2024-02-02

**Authors:** Natália P. Neme, Thomas L. C. Jansen, Remco W. A. Havenith

**Affiliations:** a Zernike Institute for Advanced Materials, University of Groningen 9747 AG Groningen The Netherlands r.w.a.havenith@rug.nl; b Stratingh Institute for Chemistry, University of Groningen 9747 AG Groningen The Netherlands; c Department of Chemistry, Ghent University Gent B-9000 Belgium

## Abstract

The aim of this study is to investigate the photophysical properties of a cyanine dye analogue by performing first-principles calculations based on density functional theory (DFT) and time dependent-DFT. Cationic cyanine dyes are the subject of great importance due to their versatile applications and the tunability of their photophysical properties, such as by modifying their end groups and chain length. An example of this is the vinylene shift, which is experimentally known for these molecules, and it consists of a bathochromic (red) shift of approximately 100 nm of the 0–0 vibronic transition when a vinyl group is added to the polymethine chain. Our study shows that when the saturated moiety C_2_H_4_ of the cyclopentene ring is added to the chain, it interacts with the conjugated π-system, resulting in a smaller HOMO–LUMO gap. Here, we demonstrate the origin of this interaction and how it can be used to fine tune the absorption energies of this class of dyes.

## Abstract 2

The aim of this study is to investigate the photophysical properties of a cyanine dye analogue by performing first-principles calculations based on density functional theory (DFT) and time dependent-DFT.

## Graphical abstract

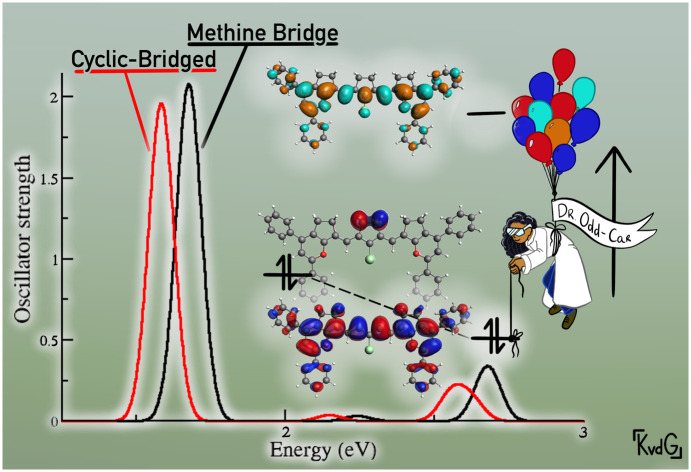

## 1 Introduction

The increasing concern related to global warming as a consequence of the intense consumption of fossil fuels worldwide and the fact that the incident global average flux of sunlight per square meter produces the energy equivalent to one petrol barrel^1^ make solar energy the most promising renewable energy source. The theoretical maximum solar energy conversion efficiency of a single junction solar cell is known as the Shockley–Queisser limit.^2^ This limit is nearly 33% for a semiconductor band gap of 1.34 eV, and it considers a balance between the particle flux in and out without any non-radiative recombination in the system, with all charge carriers being collected as current.^3^ To surpass this limit, more charge carriers must be collected. Photon up-conversion is one of the several approaches to go beyond the Shockley–Queisser limit and increase the efficiency of solar cells. Up-conversion materials are able to combine two low energy photons, typically Near Infrared (NIR) into a higher energy (UV) form.^4–8^ Zou *et al.* reported^9^ a new strategy to improve the up-conversion of lanthanide nanoparticles, which are well known up-conversion materials, by attaching dye molecules that function as antennas for the nanoparticles. The dye molecule absorbs the light, transfers the energy to the nanoparticle that performs the up-conversion, and ultimately emits one photon in the visible range. This system's concept can be broadly extended and further improved by using different dyes with different photophysical properties.

**Fig. 1:**
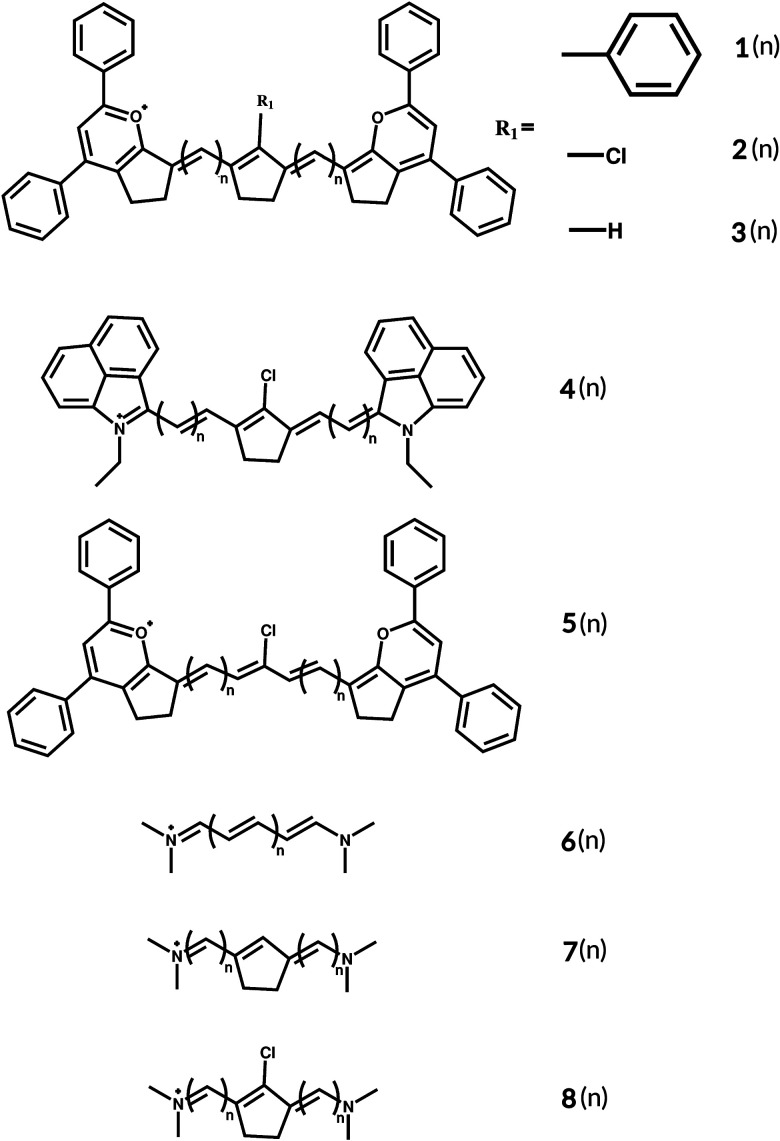
Basic structures of the studied cyanines with two different end groups and different linkers.

Cyanine dyes as used by Zou *et al.*^9^ are promising candidates for such improvement. This class of dyes is defined by the methine chain shown in Fig. 1, 6(*n*). They are versatile compounds with a wide range of applications apart from upconversion,^10–12^ such as cancer diagnosis and therapy,^13–15^ doping molecules in OLEDs,^16^ dye-sensitized solar cells (DSSEs),^17–19^ laser dyes,^20^*in vivo* imaging^21–26^ and bioimaging.^27–30^ These are cationic dyes, and they consist of two terminal groups containing nitrogen connected by a polymethine conjugated chain with 2*N* + 1 carbons. They contain an odd number of π-centres, with the π-charge density distribution alternating along the carbon chain.^31^ Charged compounds, such as cyanine dyes, have different spectroscopic properties observed in solution based on the relative importance of the dipole, bis-dipole and cyanine configurations^32^ to the electronic structure. The dipole configuration consists of a doubly degenerated dipole state where the charge is localized in one extremity. This results in a positive bond-length alternation (BLA) and symmetry breaking of the ground state, with a broad charge transfer type of transition. In the bis-dipole configuration, the charge is centrally localized in the polymethine chain, with positive BLA and a broad charge transfer transition. The cyanine configuration consists of a fully symmetric delocalized charge over the backbone, with negligible BLA and a sharp and intense transition. There are different ways to cross the cyanine limit from the cyanine to the dipolar configuration using exogenous parameters such as solvent polarity and ion-pairing. By increasing the electron donating ability of the substituent on the polymethine chain, it is possible to cross the limit from the cyanine to bis-dipole configuration.^32^

Literature data on polymethine dyes and organic molecules that absorb in the near-infrared (NIR) region are very limited^33^ and cyanine dyes are versatile candidates as a result of the different ways their photophysical properties can be tuned. The vinylene shift is known for cyanines, and it consists of a bathochromic (red) shift of approximately 100 nm of the 0–0 vibronic transition when a vinyl group is added to the polymethine chain.^31,34–37^ The particle-in-a-box model is used to explain the vinylene shift for cyanine dyes.^38^ As the heterocycles in the end groups behave as electron donors and acceptors^39^ this model restricts the electron movement to the polymethine chain with the end group being the sides of the rigid box. Different linkers on the chain can also tune the cyanine's absorption.^39^ A different approach is to choose an end group with an effective length that provides the largest red shift with the shortest polymethine chain possible, due to synthetic stability.^40^ It has been shown that in symmetric polymethine molecules, donor terminal groups considerably affect the charge distribution of the HOMO (Highest Occupied Molecular Orbital). This is a result of the mixing of the terminal groups’ wavefunctions and HOMO's conjugated chain.^41^ Not only the photophysical properties of the dye are important for practical applications, but the dye also has to be sufficiently stable. To have a more stable and rigid molecule, we can add a ring into the polymethine chain,^42^ and this ring increases the molecule's thermal and photostability,^43^ reducing the loss of energy by molecular vibrations.^44,45^ Another advantage of adding the ring is that it further stabilizes molecules that can undergo conformational changes during excitation, such as cyanines.^46^ Transitions involving π electrons that are delocalized over the conjugated system are sensitive to geometric changes in the ground and first excited states,^47^ and the central ring can prevent *cis*–*trans* photoisomerization by increasing the stability of the dye.^48^

Computational methods and the tools of theoretical chemistry can be useful to predict structure related properties, to assist the design of new dyes with specific optical properties. Cyanine dyes are still a challenge for most of the quantum chemistry methods.^49–56^ The excitation energies calculated using time-dependent density functional theory (TD-DFT) deviate up to 1 eV compared to experimental values, depending upon both the length of the polymethine chain and the end groups.^49,52,57–60^ Jacquemin *et al.*^49^ investigated disparities in vertical excitations computed using different exchange–correlation functionals and methods, by comparing them with Diffusion Monte Carlo (DMC), which is one of the most accurate methods for cyanine dyes.^52^ The study involved varying the length of the polymethine chain in the simplest cyanine with two nitrogen–hydrogen terminated chains. The results showed deviations for the GGA PBE functional in the range of 0.20–0.38 eV, for the hybrid-GGA PBE0 from 0.31 to 0.42 eV, for the long-range corrected CAM-B3LYP from 0.23 to 0.35 eV, and for the hybrid meta-GGA M05 from 0.27 to 0.38 eV, with the smallest deviation for the M06-HF functional, ranging from 0.10 to 0.21 eV. CASPT2 vertical excitations also deviate from DMC from 0.16 to 0.34 eV. Now, comparing with experiments, PBE using the Tamm–Dancoff Approximation (TDA) results in larger discrepancies ranging from 0.81 to 0.93 eV.^49^ For the double-hybrid B2PLYP functional, deviations vary from 0.64 to 0.71 eV, while PBE0 and PBE0-TDA have 0.90 to 0.99 eV and 1.43 to 1.50 eV blue-shifted vertical excitations, respectively.^52^ The trends in the vertical adiabatic TD-DFT transitions are nearly unaffected by the choice of exchange–correlation functional, with systematic overestimations compared with the experimental values;^56,60^ however, it is largely used to predict the properties of cyanine dyes.^61^ This paper presents a series of comparative studies to explore electronic, symmetry, and geometry effects on the photophysical properties of cyanine dye 1(*n*) shown in Fig. 1, which is reported to absorb at 1380 nm in 1,2-dichloroethane (DCE) and at 1408 nm in orthodichlorobenzene (ODCB).^62^ We investigated different linkers: 2(*n*) and 3(*n*) (see ESI,† Fig. S4), as well as a different end group 4(*n*) (modified FD-1080^63^) (see ESI,† Fig. S5), as shown in Fig. 1. Aiming to explore the photophysical properties of this dye, we varied the polymethine chain's length of 2(*n*) from *n* = 1 to 8.

The goal of this study is to provide an understanding of possible ways to tune the absorption of this class of dyes as candidates to be used as antennas on a lanthanide-based inorganic–organic hybrid system to perform upconversion and improve the PCEs of solar cells. The findings of the current study indicate that the methine bridge substituents play an important role, not only for their stability. We will further elucidate the mechanism behind the observed effects.

## 2 Computational details

Density Functional Theory (DFT)^64,65^ and TD-DFT^66^ calculations were performed using the Amsterdam Modelling Suite (AMS)^67^ (and ORCA 5.0.4^68,69^). All DFT and TD-DFT calculations were performed using the hybrid B3LYP^70^ exchange–correlation functional in combination with the TZP basis set.^71,72^ A more elaborated study of the functional and basis set dependence is presented in the ESI.† All functionals (including range-separated hybrids CAM-B3LYP,^73^ ωB97X^74^ and the double-hybrid functional B2PLYP^75^) and basis sets (AUG/ATZP^76^ and def2-TZVP/de2-TZVP/C^77^) show the same features as obtained by B3LYP/TZP. Hence, we discuss the B3LYP/TZP numbers in the main text, and the observed trends are independent of the choice of functional and basis set. The geometries were optimized and the vibrational normal modes and frequencies were calculated to characterize the nature of the stationary points, confirming that the ground states correspond to true minima of the potential energy surface. To evaluate the solvent effect, orthodichlorobenzene (ODCB) was used as a solvent with the Minnesota Solvation Model 12 (SM12).^78^ All calculations were performed using Becke 3 (good)^79^ for geometry optimizations and UV-vis spectra without any symmetry constraints. The UV-vis spectra were convoluted using Gaussian functions having 0.1 eV of half width at half-maximum (HWHM) and they were generated using the graphical tool Xmgrace.^80^ The geometries of all described molecules are given in the ESI.† The Natural Bond Orbital (NBO) analysis was performed to determine the interaction between different fragments of the molecule using NBO 6.0.^81^ The procedure consisted of several steps: the first one was obtaining the Fock matrix in the NBO basis, as provided by the NBO 6.0 program. Molecules 2(*n*) (*n* = 1 and 2) were divided into two fragments: the C_2_H_4_ saturated moiety of the ring on the polymethine chain and the remaining of the molecule (see Fig. 6 in Results and discussion). All NBOs were assigned to either fragment. To ‘switch off’ the interaction between the two fragments, the Fock matrix elements between NBOs assigned to different fragments were set to zero. Diagonalization of this new Fock matrix resulted in a set of semi-canonical molecular orbitals delocalized over one of the fragments. The Fock matrix was subsequently transformed into the basis of this set of semi-canonical molecular orbitals; the off-diagonal elements of this Fock matrix are indicative of the interactions between orbitals centred at different fragments, and the diagonal Fock matrix elements can be interpreted as orbital energies of the semi-canonical molecular orbitals, in case there is no interaction between the fragments.

To validate that the TD-DFT results are also reproduced with more accurate wavefunction methods, the second-order approximate coupled-cluster model (CC2) using the resolution of the identity (RI) approximation^82^ was used to compute the vertical excitation energy for a subset of molecules 6(*n*) and 8(*n*) using Turbomole 7.6^83^ and the def-TZVP basis set.^77^

## 3 Results and discussion

**Fig. 2:**
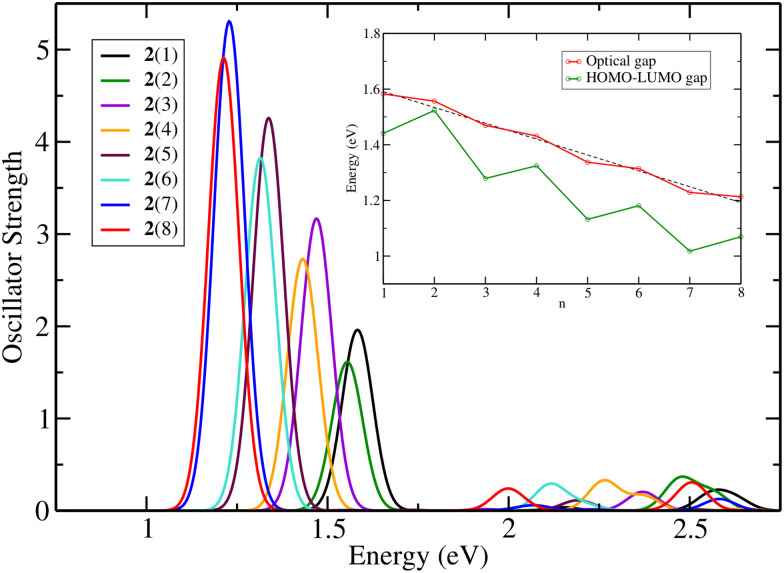
Theoretical absorbance spectra of molecules 2(*n*) with *n* = 1 to 8 in the gas phase at the TD-DFT/B3LYP/TZP level with 5 excited states. The inset graph shows the optical and the HOMO–LUMO gaps.

Fig. 2 shows the theoretical absorbance spectra of molecules 2(*n*) (Fig. 1) obtained by varying the length of the chain. Here, we define *n* as the number of C–H added to each side of the chain. The total number of carbons added is always 2*n*. It can be seen that the expected periodic 100 nm vinylene shift for cyanine dyes is not applicable. Another important finding is that the molecules can be divided into two groups: one consisting of the molecules with *n* = odd carbons added to each side of the ring and one with *n* = even carbon atoms added. Because of the central ring and to keep the symmetry of the molecule, *n* is the number of C–H added to each side. The main absorption peak for all molecules corresponds to a HOMO–LUMO (Highest Occupied Molecular Orbital–Lowest Unoccupied Molecular Orbital) transition in the first excited state. The inset graph in Fig. 2 provides an overview of the optical (*E*_exc_) and HOMO–LUMO gaps (*E*_H–L_), and it can be seen that the latter has a similar trend in a non-linear decrease in the optical gap with the increase of the number of carbons in the polymethine chain. The linear regression shows the trend for the optical gap, even though being less noticeable than the HOMO–LUMO gap. The coefficient of determination for molecules 2(*n*) between *E*_H–L_ and *E*_exc_ is 0.91 (see Fig. S1, ESI†), while for molecules 6(*n*) and 8(*n*) the coefficients are 1.00 and 0.99, respectively (see Fig. S2, ESI†). The addition of the cyclopentene ring to the polymethine chain reduces the HOMO–LUMO gap, as does the presence of more complex end groups in the molecule. Despite this reduction, the correlation of 0.91 for molecules 2(*n*) indicates that changes in the optical gap can be analysed based on changes in the HOMO–LUMO gap.

Photoisomerization in cyanine dyes, despite garnering increasing interest in recent years, remains a highly elusive phenomenon that is strongly influenced by local environmental factors, such as solvent polarity and temperature.^84,85^ Through conformation analysis of molecules 2(*n*), it has been determined that molecules belonging to the first group (*n* = odd) adopt a *trans*–*trans* configuration, exhibiting an energy difference of approximately 5 kcal mol^−1^ between the *trans*–*trans* (Fig. S7(a), ESI†) and *trans*–*cis* (Fig. S7(b), ESI†) conformers. Conversely, the second group (*n* = even) adopts a *trans*–*cis* configuration, with a smaller energy difference of approximately 3 kcal mol^−1^. Interestingly, despite the distinct conformations, the primary absorption peak for both conformers does not exhibit a significant shift, with a maximum shift of 0.018 eV for the first group and 0.035 eV for the second group. Moreover, both the energy difference and absorption shift between the conformers decrease as the length of the backbone chain increases (see Fig. S8 and S9, ESI†).

**Fig. 3:**
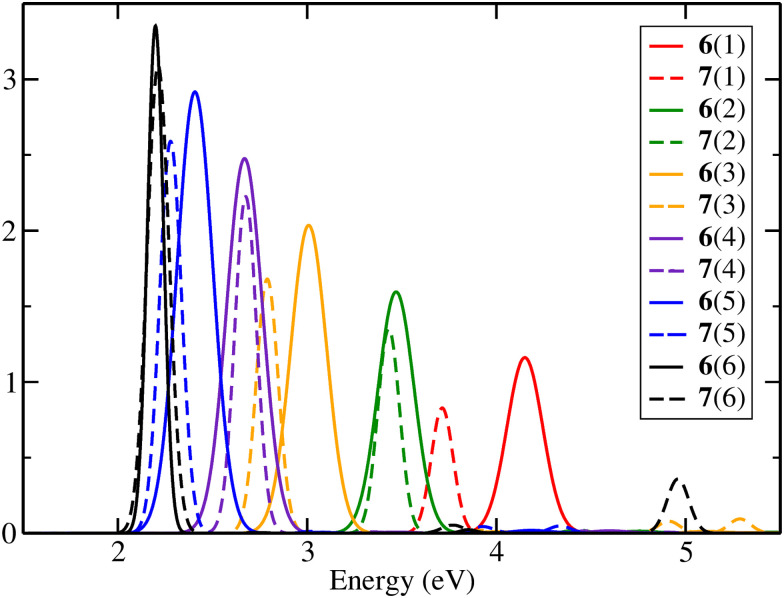
Theoretical absorbance spectra of molecules 6(*n*) and 7(*n*) with *n* = 1 to 6 in the gas phase at the TD-DFT/B3LYP/TZP level with 5 excited states.

An alternating behaviour was observed for the oscillator strength of the first transition for molecules 2(*n*). Fig. 3 provides a comparison of the absorption spectra of molecules 6(*n*) and 7(*n*), revealing no alternation in the intensity. Therefore, it can be inferred that this variation is attributed to the distinct end groups present in the molecules, not an effect of the ring added to the backbone chain. The oscillator strength is affected as a result of the mixing between the terminal groups’ and the chain's MOs.

As discussed before, the TD-DFT excitation energy tends to deviate from experimental values. In order to validate the finding using a more accurate wavefunction method, second-order approximate Coupled-Cluster (CC2) calculations^82^ were performed on molecules 6(*n*) and 8(*n*) with *n* = 1 to 4. Tables S1 and S2 (ESI†) show a comparison between the vertical excitation from the ground state S_0_ to the first excited state S_1_. Besides the difference in absolute values compared with TD-DFT, CC2 has similar shifts, and this provides a confirmation of our findings based on the TD-DFT calculations.

Solvation effects are predominantly governed by the alterations in molecular dipole moments upon excitation, which encompass two primary factors: the dipole transition change during excitation and the disparity between the permanent dipole moments of the ground and excited states, both of which are well-established for cyanine dyes.^86^ In Fig. S6 (ESI†), the absorption spectra considering implicit solvation effects are presented, revealing negligible solvatochromism. It is widely recognized that cationic dyes exhibit varying shifts contingent upon the polarization of the solvent. However, the introduction of the central ring into the polymethine chain can effectively suppress this solvatochromism to a significant extent.^45,87^

**Fig. 4:**
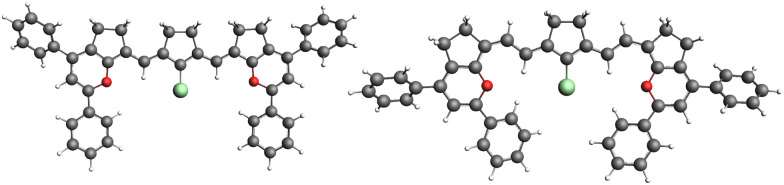
Optimized ground state geometries of molecules 2(1) and 2(2) are shown on the left and right sides, respectively.

Molecules 3(*n*) (Fig. S4, ESI†) with *n* = 1 to 4 and a hydrogen as a linker also show no periodic vinylene shift, as well as molecules 4(*n*) with a different end group (Fig. S5, ESI†). The exchange of linker R_1_ from chlorine 2(*n*) (Fig. 4) to hydrogen 3(*n*) results in a small blue shift in the absorption for all molecules, keeping the non-periodic vinylene shift. This indicates that linker R_1_ and the end groups are not associated with the non-periodicity of the absorption shift and the failure of the particle-in-a-box model for the optical gap of this dye.

**Fig. 5:**
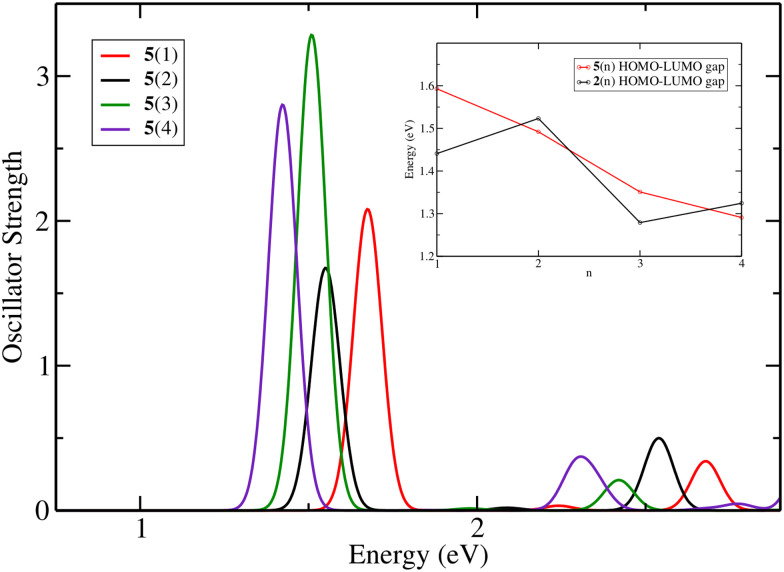
Theoretical absorbance spectra of molecules 5(*n*) with *n* = 1 to 4 in the gas phase at the TD-DFT/B3LYP/TZP level with 5 excited states. The inset graph shows a comparison between the HOMO–LUMO gap for 2(*n*) and 5(*n*).

To investigate whether the saturated ring plays a role in this behaviour, we studied molecules 5(*n*), which are similar to 1(*n*), but are lacking the five-membered ring. Fig. 5 shows the absorption spectra of 5(*n*) with *n* varying from 1 to 4, and it can be seen that by removing the ring we essentially retrieve the periodic shift expected by the particle-in-a-box model; hence, the ring affects the absorption. By adding cyclopentene to the polymethine chain, the absorption of molecules 5(1) and 5(3) red shifted by 0.09 and 0.04 eV, respectively. For molecules 5(2) and 5(4) the blue shift is negligible, being 0.004 and 0.008 eV, respectively. In fact, the HOMO–LUMO gap for the group *n* = odd carbons is more affected by the ring than that for the group *n* = even carbons, as shown in the inset graph in Fig. 5. The molecule *n* = 1 has a HOMO (LUMO) energy of −6.87 (−5.41) eV with the ring, and by removing C_2_H_4_ the HOMO energy changes to −7.02 eV and the LUMO to −5.43 eV. The variation in HOMO–LUMO energy for the *n* = 2 molecule is smaller, with the HOMO varying from −6.89 to −6.94 eV and the LUMO from −5.36 to −5.44 eV. Taken together, these results suggest that there is an association between the polymethine chain's ring and the fact that the particle-in-a-box model cannot explain the absorption shift for this molecule.

**Fig. 6:**
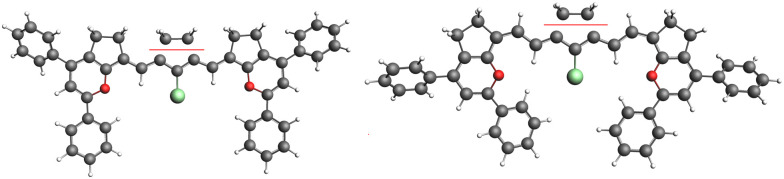
The two fragments of molecules 2(*n*): *n* = 1 and *n* = 2 for the NBO study in which the interaction between C_2_H_4_ and the polymethine chain was set to zero.

Surprisingly, despite being saturated, the ring affects the absorption maximum and not only the stability of the molecule, as previously reported.^44^ To further understand the reason how the ring has different effects on the two groups of molecules (*n* = odd and even) we performed NBO analysis. The analysis consisted of calculating the orbital energies of the molecules, but neglecting interactions between the C_2_H_4_ moiety and the polymethine chain (see Fig. 6). Since the HOMO–LUMO gap follows the same pattern as the optical gap and the HOMO–LUMO transition is the main transition to the first excited state, the analysis was done on the orbital energies.

**Fig. 7:**
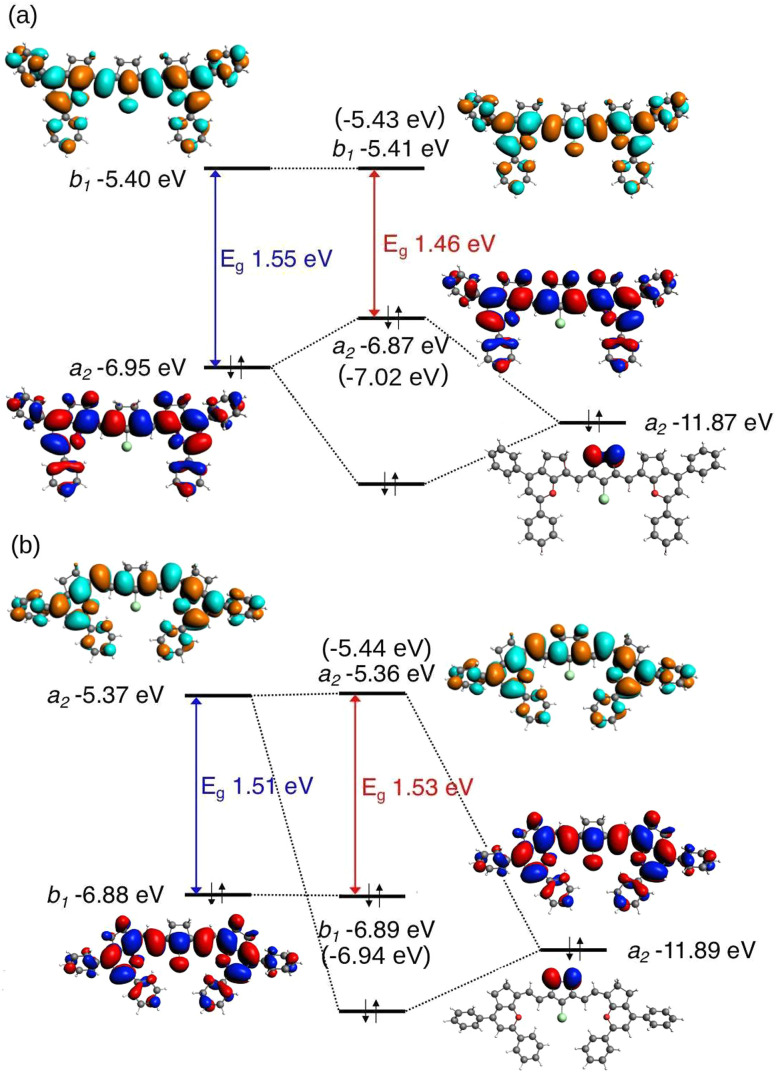
Saturated ring analysis of molecules 2(*n*): *n* = 1 (a) and *n* = 2 (b), NBO study excluding interaction between C_2_H_4_ and the polymethine chain. The orbital energies of the molecules without the C_2_H_4_ moiety (5(*n*)) are presented in brackets. HOMOs are presented in blue-red and the LUMOs are presented in cyan-orange. The colour difference in the molecular orbitals indicates a difference in sign.

Fig. 7 compares the HOMO and LUMO with and without the interactions. Considering the *C*_2v_ symmetry point group, for the molecules with *n* = odd carbons added to the chain, the HOMO transforms according to the *a*_2_ irreducible representation and the LUMO according to the *b*_1_ irreducible representation. For the molecules in the second group, *n* = even, the symmetries of the frontier orbitals are inverted. The C–H bonding orbitals on the C_2_H_4_ moiety form four symmetry adapted linear combinations, of which the one with *a*_2_ symmetry has the highest energy. In Fig. 7(a), it can be seen that for molecule 2(1) the *a*_2_ combination of the C–H bonding orbitals with an energy of −11.87 eV and symmetry *a*_2_ interact with the HOMO with an energy of −6.95 eV, thereby destabilizing the HOMO and resulting in a reduction of the HOMO–LUMO gap from 1.55 eV to 1.46 eV. Because of the inversion of symmetry of the HOMO and LUMO when a vinylene group is added to the chain, for molecule 2(2) the *a*_2_ C–H combination of the C_2_H_4_ moiety with an energy of −11.89 eV can interact with the LUMO and not with the HOMO. This interaction has no considerable effect on the LUMO energy because of the larger energy gap and weaker interaction.

## 4 Conclusion

We performed DFT and TD-DFT calculations to study the vinylene shift of a cyanine analogue containing a cyclopentene in the methine bridge. This study has shown that the chain's ring affects not only the stability of the dye, as reported experimentally,^44^ but also the photophysical properties. These properties can be tuned as a result of the interaction between the moiety and the polymethine chain, depending on the number of carbon atoms in the chain. For molecules with *n* = odd carbons added to the chain, the HOMO that transforms according to the *a*_2_ representation interacts with the *a*_2_ combination of C–H orbitals of the C_2_H_4_ moiety, increasing the HOMO energy and reducing the HOMO–LUMO gap. For molecules with *n* = even carbons added to the chain, the HOMO has symmetry *b*_1_ and therefore does not interact with the C_2_H_4_ orbitals in the ring. The results of this study suggest that the substitutions to the methine bridge can affect the photophysical properties of molecules, depending on the number of carbons and the symmetry of the frontier orbitals. We find that this property is independent of the cyanine's end groups and linker substitutions, suggesting that it is potentially relevant for other polymethine dyes with bridges. The present work provides an additional method for tuning the photophysical properties of cyanine dyes, which is an important tool for designing new materials.

## Data availability

The data underlying this study are available in the published article and its ESI.†

## Conflicts of interest

There are no conflicts to declare.

## Supplementary Material

CP-026-D3CP05219B-s001

CP-026-D3CP05219B-s002

## References

[cit1] Council W. E. , World energy resources solar 2016, 2016

[cit2] Shockley W., Queisser H. J. (1961). J. Appl. Phys..

[cit3] Xu Y., Gong T., Munday J. N. (2015). Sci. Rep..

[cit4] Singh-Rachford T. N., Castellano F. N. (2010). Coord. Chem. Rev..

[cit5] Weingarten D. H., LaCount M. D., van de Lagemaat J., Rumbles G., Lusk M. T., Shaheen S. E. (2017). Nat. Commun..

[cit6] Auzel F. (2004). Chem. Rev..

[cit7] Wang H., Xu Y., Pang T., Chen B., Xin F., Xing M., Tian M., Fu Y., Luo X., Tian Y. (2022). Nanoscale.

[cit8] Patel M., Meenu M., Pandey J. K., Kumar P., Patel R. (2022). J. Rare Earths.

[cit9] Zou W., Visser C., Maduro J. A., Pshenichnikov M. S., Hummelen J. C. (2012). Nat. Photonics.

[cit10] Shindy H. (2017). Dyes Pigms..

[cit11] Benson R. C., Kues H. A. (1977). J. Chem. Eng. Data.

[cit12] Ma X., Shi L., Zhang B., Liu L., Fu Y., Zhang X. (2022). Anal. Bioanal. Chem..

[cit13] Komljenovic D., Wiessler M., Waldeck W., Ehemann V., Pipkorn R., Schrenk H.-H., Debus J., Braun K. (2016). Theranostics.

[cit14] Stummer W., Stocker S., Novotny A., Heimann A., Sauer O., Kempski O., Plesnila N., Wietzorrek J., Reulen H. (1998). J. Photochem. Photobiol., B.

[cit15] Bai L., Hu Z., Han T., Wang Y., Xu J., Jiang G., Feng X., Sun B., Liu X., Tian R. (2022). et al.. Theranostics.

[cit16] Shoustikov A. A., You Y., Thompson M. E. (1998). IEEE J. Sel. Top. Quantum Electron..

[cit17] Narayan M. R., Sust Renew (2012). Energy Rev..

[cit18] Zhou H., Wu L., Gao Y., Ma T. (2011). J. Photochem. Photobiol., A.

[cit19] Castro F. A., Faes A., Geiger T., Graeff C. F., Nagel M., Nüesch F., Hany R. (2006). Synth. Met..

[cit20] Czerney P., Graneß G., Birckner E., Vollmer F., Rettig W. (1995). J. Photochem. Photobiol., A.

[cit21] Šmidlehner T., Kurutos A., Slade J., Belužić R., Ang D. L., Rodger A., Piantanida I. (2018). Eur. J. Org. Chem..

[cit22] Klohs J., Baeva N., Steinbrink J., Bourayou R., Boettcher C., Royl G., Megow D., Dirnagl U., Priller J., Wunder A. (2009). J. Cereb..

[cit23] Kurutos A., Orehovec I., Paić A. T., Crnolatac I., Horvat L., Gadjev N., Piantanida I., Deligeorgiev T. (2018). Dyes Pigms..

[cit24] Kurutos A., Ilic-Tomic T., Kamounah F. S., Vasilev A. A., Nikodinovic-Runic J. (2020). J. Photochem. Photobiol., A.

[cit25] Abeywickrama C. S. (2022). Chem. Commun..

[cit26] Higuchi K., Sato Y., Togashi N., Suzuki M., Yoshino Y., Nishizawa S. (2022). ACS Omega.

[cit27] Medeiros N. G., Braga C. A., Câmara V. S., Duarte R. C., Rodembusch F. S. (2022). Asian J. Org. Chem..

[cit28] Escobedo J. O., Rusin O., Lim S., Strongin R. M. (2010). Curr. Opin. Chem. Biol..

[cit29] Tan X., Constantin T. P., Sloane K. L., Waggoner A. S., Bruchez M. P., Armitage B. A. (2017). J. Am. Chem. Soc..

[cit30] Guo Z., Nam S., Park S., Yoon J. (2012). Chem. Sci. J..

[cit31] Mustroph H. (2020). Phys. Sci. Rev..

[cit32] Pascal S., Haefele A., Monnereau C., Charaf-Eddin A., Jacquemin D., Le Guennic B., Andraud C., Maury O. (2014). J. Phys. Chem. A.

[cit33] Fabian J., Nakazumi H., Matsuoka M. (1992). Chem. Rev..

[cit34] Mustroph H. (2019). Phys. Sci. Rev..

[cit35] Kachkovski A., Kovalenko N. (1997). Dyes Pigms..

[cit36] Ishchenko A., Derevyanko N., Zubarovskii V., Tolmachev A. (1984). Theor. Exp. Chem..

[cit37] Tolmachev A., Romanov N., Fedotov K., Dyadyusha G., Kachkovski A. (1988). Dyes Pigms..

[cit38] Autschbach J. (2007). J. Chem. Educ..

[cit39] Levitz A., Marmarchi F., Henary M. (2018). Photochem. Photobiol. Sci..

[cit40] Tolmachev A. , Slominskii Y. L. and Ishchenko A., in Near-infrared dyes for high technology applications, Springer, 1998, pp. 385–415

[cit41] Ooyama Y., Harima Y. (2009). Eur. J. Org. Chem..

[cit42] Henary M. and Mojzych M., Heterocyclic polymethine dyes: synthesis, properties and applications, 2008, pp. 221–238

[cit43] Przhonska O. V., Hu H., Webster S., Bricks J. L., Viniychuk A. A., Kachkovski A. D., Slominsky Y. L. (2013). Chem. Phys..

[cit44] Mohammad I., Stanford C., Morton M. D., Zhu Q., Smith M. B. (2013). Dyes Pigms..

[cit45] Bertolino C. A., Ferrari A. M., Barolo C., Viscardi G., Caputo G., Coluccia S. (2006). Chem. Phys..

[cit46] Dietz F., Rentsch S. (1985). Chem. Phys..

[cit47] Berlman I. B. (1970). J. Phys. Chem..

[cit48] Pronkin P., Tatikolov A. (2019). Science.

[cit49] Jacquemin D., Zhao Y., Valero R., Adamo C., Ciofini I., Truhlar D. G. (2012). J. Chem. Theory Comput..

[cit50] Galindo L., Gomes O., Graeff C., Batagin-Neto A. (2021). Comput. Theor. Chem..

[cit51] Fabian J. (2010). Dyes Pigms..

[cit52] Send R., Valsson O., Filippi C. (2011). J. Chem. Theory Comput..

[cit53] Meguellati K., Ladame S., Spichty M. (2011). Dyes Pigm..

[cit54] Champagne B., Guillaume M., Zutterman F. (2006). Chem. Phys. Lett..

[cit55] Schreiber M., Buß V., Fülscher M. P. (2001). Phys. Chem. Chem. Phys..

[cit56] Le Guennic B., Jacquemin D. (2015). Acc. Chem. Res..

[cit57] Charaf-Eddin A., Le Guennic B., Jacquemin D. (2014). RSC Adv..

[cit58] Minezawa N. (2015). Chem. Phys. Lett..

[cit59] Laurent A. D., Adamo C., Jacquemin D. (2014). Phys. Chem. Chem. Phys..

[cit60] Zhekova H., Krykunov M., Autschbach J., Ziegler T. (2014). J. Chem. Theory Comput..

[cit61] Matikonda S. S., Hammersley G., Kumari N., Grabenhorst L., Glembockyte V., Tinnefeld P., Ivanic J., Levitus M., Schnermann M. J. (2020). J. Org. Chem..

[cit62] Drexhage K. H. , Krussler M., Sens B. and Marx M. J., Deutsches Pat., DE3316666C2, 1984

[cit63] Sun C., Li B., Zhao M., Wang S., Lei Z., Lu L., Zhang H., Feng L., Dou C., Yin D. (2019). et al.. J. Am. Chem. Soc..

[cit64] Hohenberg P., Kohn W. (1964). Phys. Rev..

[cit65] Kohn W., Sham L. J. (1965). Phys. Rev..

[cit66] Runge E., Gross E. K. (1984). Phys. Rev. Lett..

[cit67] T. N. Vrije Universiteit, Amsterdam, *AMS 2022.1*, *SCM*, *Theoretical Chemistry*, https://www.scm.com

[cit68] Neese F., Wennmohs F., Becker U., Riplinger C. (2020). J. Chem. Phys..

[cit69] Neese F. (2022). Wiley Interdiscip. Rev.: Comput. Mol. Sci..

[cit70] Stephens P. J., Devlin F. J., Chabalowski C. F., Frisch M. J. (1994). J. Phys. Chem..

[cit71] Van Lenthe E., Baerends E. J. (2003). J. Comput. Chem..

[cit72] Chong D. P., Van Lenthe E., Van Gisbergen S., Baerends E. J. (2004). J. Comput. Chem..

[cit73] Yanai T., Tew D. P., Handy N. C. (2004). Chem. Phys. Lett..

[cit74] Chai J.-D., Head-Gordon M. (2008). J. Chem. Phys..

[cit75] Grimme S. (2006). J. Chem. Phys..

[cit76] Chong D. (2005). Mol. Phys..

[cit77] Schäfer A., Huber C., Ahlrichs R. (1994). Chem. Phys..

[cit78] Marenich A. V., Cramer C. J., Truhlar D. G. (2013). J. Chem. Theory Comput..

[cit79] Franchini M., Philipsen P. H. T., Visscher L. (2013). J. Comput. Chem..

[cit80] Turner P. , Center for Coastal and Land-Margin Research, 2005, vol. 2

[cit81] Glendening E. D., Landis C. R., Weinhold F. (2013). J. Comput. Chem..

[cit82] Christiansen O., Koch H., Jørgensen P. (1995). Chem. Phys. Lett..

[cit83] Ahlrichs R., Bär M., Häser M., Horn H., Kölmel C. (1989). Chem. Phys. Lett..

[cit84] Sandberg E., Piguet J., Kostiv U., Baryshnikov G., Liu H., Widengren J. (2023). J. Phys. Chem. B.

[cit85] Mishra A., Behera R. K., Behera P. K., Mishra B. K., Behera G. B. (2000). Chem. Rev..

[cit86] McRae E. (1957). J. Phys. Chem..

[cit87] Yu A., Tolbert C. A., Farrow D. A., Jonas D. M. (2002). J. Phys. Chem. A.

