# Supplementary material for: Cyclopentene ring effects in cyanine dyes: a handle to fine-tune photophysical properties
Source: Phys Chem Chem Phys. 2024 Feb 2;26(7):6235–41. doi: 10.1039/d3cp05219b (PMC10866127; doi:10.1039/d3cp05219b)
Supplement: CP-026-D3CP05219B-s001 [file CP-026-D3CP05219B-s001.pdf]

Electronic Supplementary Material (ESI) for Physical Chemistry Chemical Physics.
This journal is © the Owner Societies 2024

Supporting Information:

Cyclopentene ring effects in cyanine dyes: a handle to fine-tune photophysical properties

Natalia P. Neme [1,2], Thomas L.C Jansen [1], and Remco W.A. Havenith [1,2,3⋆]

[1] *Zernike Institute for Advanced Materials, University of Groningen, 9747 AG Groningen, The Netherlands*

[2] *Stratingh Institute for Chemistry, University of Groningen, 9747 AG Groningen, The Netherlands*

[3] *Department of Chemistry, Ghent University, B-9000 Gent, Belgium*

⋆ Email: r.w.a.havenith@rug.nl

Contents

List of Figures **S-2**

1 Coefficient of determination for molecules 2(n) **S-5**

2 Coefficient of determination for molecules 6(n) and 8(n) **S-6**

3 Theoretical absorbance at CC2 and TD-DFT **S-7**

4 Theoretical absorbance spectra for molecules 3(n) and 4(n) **S-8**

5 Theoretical absorbance spectra for 2(n) with implicit solvation **S-10**

6 Conformation analysis **S-13**

7 Benchmark **S-18**

7.1 ωB97X/TZP S-18

7.2 CAM-B3LYP/TZP S-21

7.3 B2PLYP/def2-TZVP/def2-TZVP/C S-23

7.4 B3LYP/AUG/ATZP S-27

7.5 ωB97X/AUG/ATZP S-29

8 Vertical TD-DFT excitation and main orbitals electronic transitions for molecules 1-8(n) in gas phase at B3LYP/TZP level **S-32**

List of Figures

S1 Linear regression and coefficient of determination for molecules **2**(n) with n=1 to 8 in gas phase at TD-DFT/B3LYP/TZP S-5

S2 Linear regression and coefficient of determination for molecules **6**(n) (blue) and **8**(n) (red) with n=1 to 6 in gas phase at TD-DFT/B3LYP/TZP S-6

S3 Vertical excitation transitions S0-S1 for molecules **6**(n) and **8**(n) with n=1 to 6. The S0 were optimized using at DFT level using B3LYP/TZP. CC2 transitions were calculated with def-TZVP basis set S-7
S4 Theoretical absorbance spectra for molecules **3**(n) with n=1 to 4 in gas phase at TD-DFT/B3LYP/TZP level with 5 excited states. The inset shows the basic structure for the molecule. S-8
S5 Theoretical absorbance spectra for molecules **4**(n) with n=1 to 4 in gas phase at TD-DFT/B3LYP/TZP level with 5 excited states. The inset shows the basic structure for the molecule. S-9
S6 Theoretical absorbance spectra for molecules **2**(n) with n=1 to 4 in solvent ODCB at TD-DFT/B3LYP/TZP level with 5 excited states. The inset shows the basic structure for the molecule. S-10
S7 (a) trans-trans and (b) trans-cis structure for molecules **2**(n). S-13
S8 Energy difference between different conformers. For the first group (n=odd), trans-trans conformer is more stable. For the second group (n=even), trans-cis conformer is more stable. S-13
S9 Main absorption peak for all trans-trans and trans-cis conformers . . S-14
S10 Theoretical absorbance spectra for molecules **8**(n) with n=1 to 6 in gas-phase at TD-DFT/ω97BX/TZP level with 5 excited states. . . . S-18
S11 Theoretical absorbance spectra for molecules **8**(n) with n=1 to 6 in gas-phase at TD-DFT/CAM-B3LYP/TZP S-21
S12 Theoretical absorbance spectra for molecules **6**(n) with n=1 to 6 in gas-phase at TD-DFT/B2PLYP/def2-TZVP/def2-TZVP/C S-23
S13 Theoretical absorbance spectra for molecules **8**(n) with n=1 to 6 in gas-phase at TD-DFT/B2PLYP/def2-TZVP/def2-TZVP/C S-25
S14 Theoretical absorbance spectra for molecules **8**(n) with n=1 to 6 in gas-phase at TD-DFT/B3LYP/AUG/ATZP S-27

S15 Theoretical absorbance spectra for molecules **8**(n) with n=1 to 6 in gas-phase at TD-DFT/ωB97X/AUG/ATZP S-29

1 Coefficient of determination for molecules 2(n)

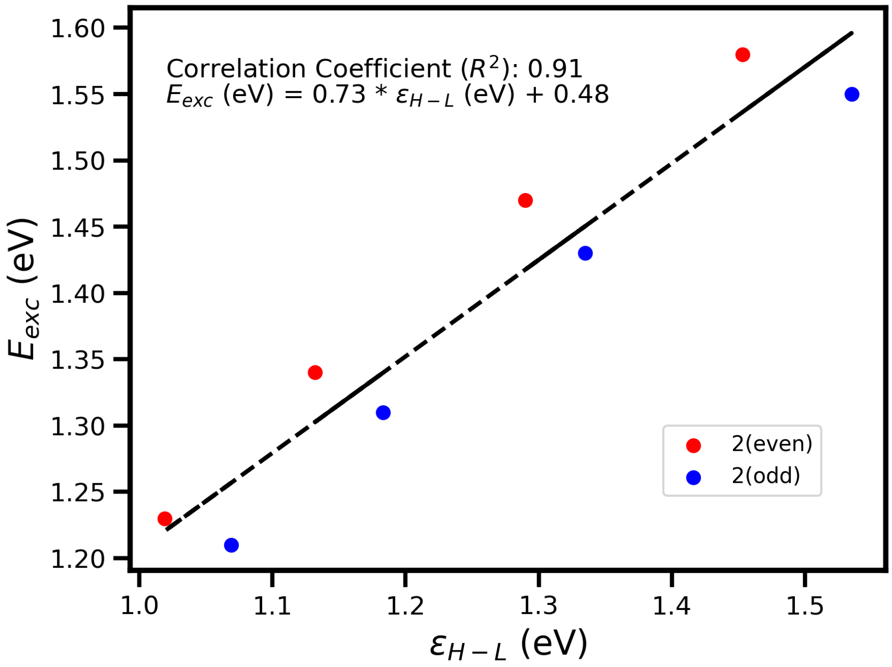

Figure S1: Linear regression and coefficient of determination for molecules **2**(n) with n=1 to 8 in gas phase at TD-DFT/B3LYP/TZP

2 Coefficient of determination for molecules 6(n) and 8(n)

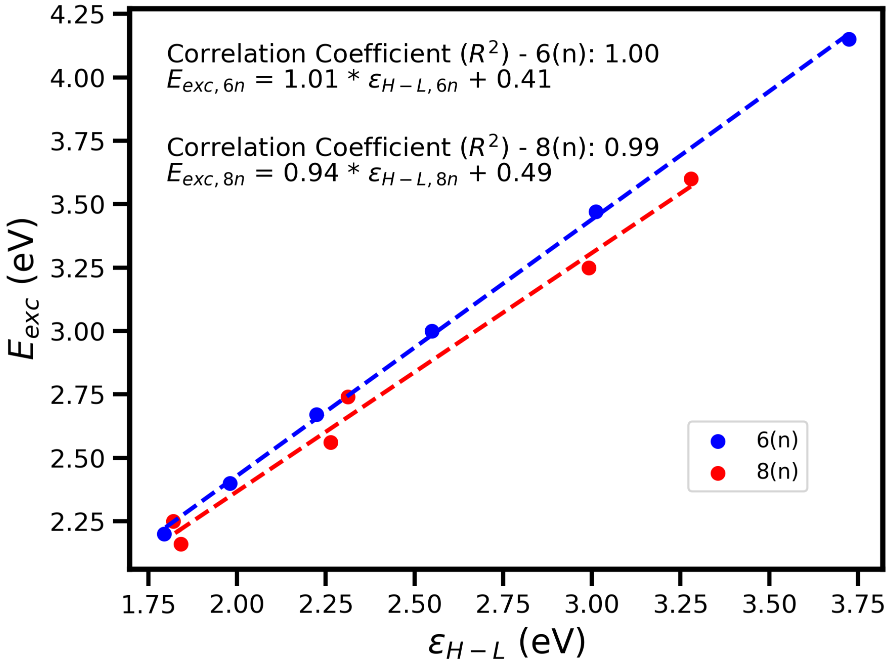

Figure S2: Linear regression and coefficient of determination for molecules **6**(n) (blue) and **8**(n) (red) with n=1 to 6 in gas phase at TD-DFT/B3LYP/TZP

3 Theoretical absorbance at CC2 and TD-DFT

Table S1: Table comparing the vertical excitation transitions S0-S1 at CC2 and TD-DFT level for molecules **8**(n) with n=1 to 4. For both calculations, the S0 were optimized using at DFT level using B3LYP/TZP. CC2 transitions were calculated with def-TZVP basis set, and TD-DFT using B3LYP/TZP.

	**CC2 (eV)**	f_{osc}	E_{exc} **(eV)**	f_{osc}
**6(1)**	3.72	1.33	4.15	1.16
**6(2)**	3.03	1.81	3.47	1.59
**6(3)**	2.56	1.93	3.00	2.03
**6(4)**	2.24	2.78	2.67	2.47

Table S2: Table comparing the vertical excitation transitions S0-S1 at CC2 and TD-DFT level for molecules **8**(n) with n=1 to 4. For both calculations, the S0 were optimized using at DFT level using B3LYP/TZP. CC2 transitions were calculated with def-TZVP basis set, and TD-DFT using B3LYP/TZP.

	**CC2 (eV)**	f_{osc}	E_{exc} **(eV)**	f_{osc}
**8(1)**	3.32	0.95	3.60	0.74
**8(2)**	2.91	1.46	3.25	1.11
**8(3)**	2.41	1.93	2.74	1.57
**8(4)**	2.20	2.45:	2.56	2.06

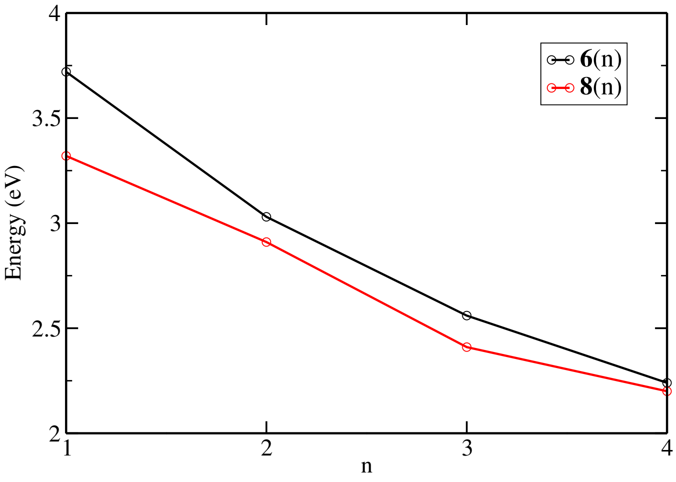

Figure S3: Vertical excitation transitions S0-S1 for molecules **6**(n) and **8**(n) with n=1 to 6. The S0 were optimized using at DFT level using B3LYP/TZP. CC2 transitions were calculated with def-TZVP basis set

4 Theoretical absorbance spectra for molecules 3(n) and 4(n)

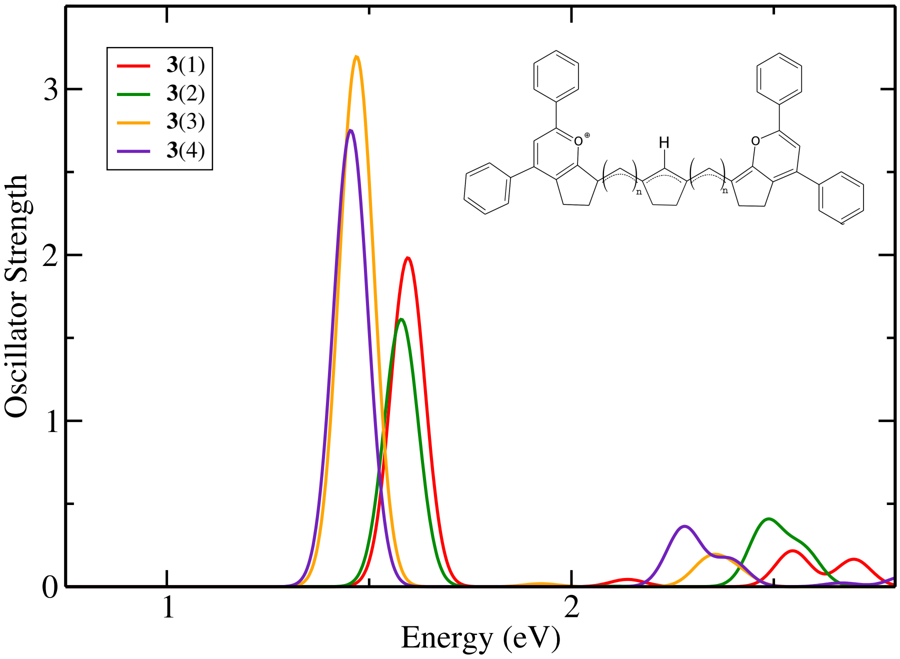

Figure S4: Theoretical absorbance spectra for molecules **3**(n) with n=1 to 4 in gas phase at TD-DFT/B3LYP/TZP level with 5 excited states. The inset shows the basic structure for the molecule.

Table S3: Vertical excitation transitions S0-S1 for molecule **3**(n) with n=1 to 4 at TD-DFT/B3LYP/TZP.

	E_{exc} (eV)	f_{osc}
**3(1)**	1.59	1.98
**3(2)**	1.58	1.61
**3(3)**	1.47	3.19
**3(4)**	1.45	2.75

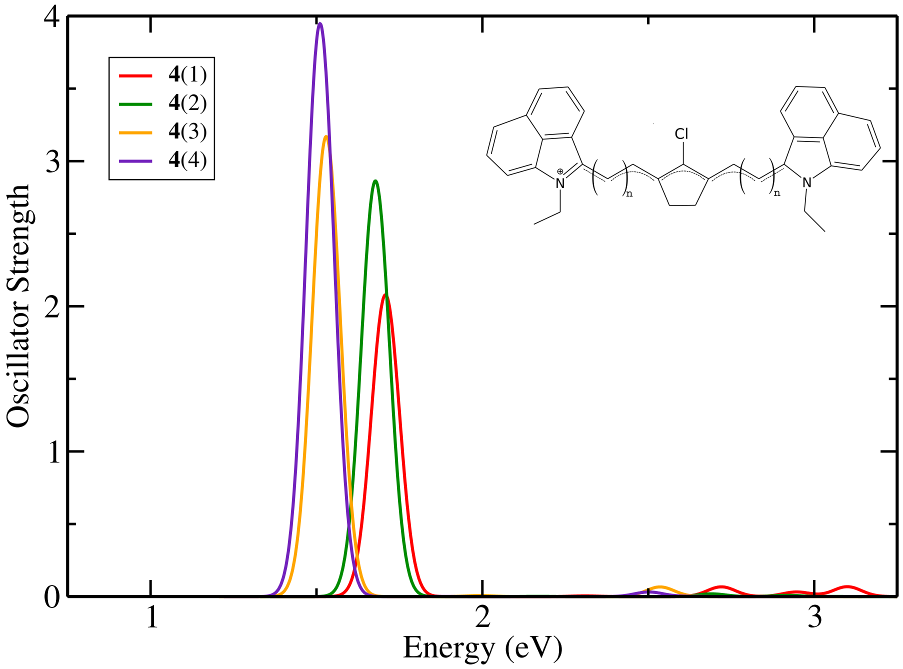

Figure S5: Theoretical absorbance spectra for molecules **4**(n) with n=1 to 4 in gas phase at TD-DFT/B3LYP/TZP level with 5 excited states. The inset shows the basic structure for the molecule.

Table S4: Vertical excitation transitions S0-S1 for molecules **4**(n) with n=1 to 4 in solvent ODCB at TD-DFT/B3LYP/TZP.

	E_{exc} **(eV)**	f_{osc}
**4(1)**	1.71	2.07
**4(2)**	1.68	2.86
**4(3)**	1.53	3.17
**4(4)**	1.51	3.94

5 Theoretical absorbance spectra for 2(n) with implicit solvation

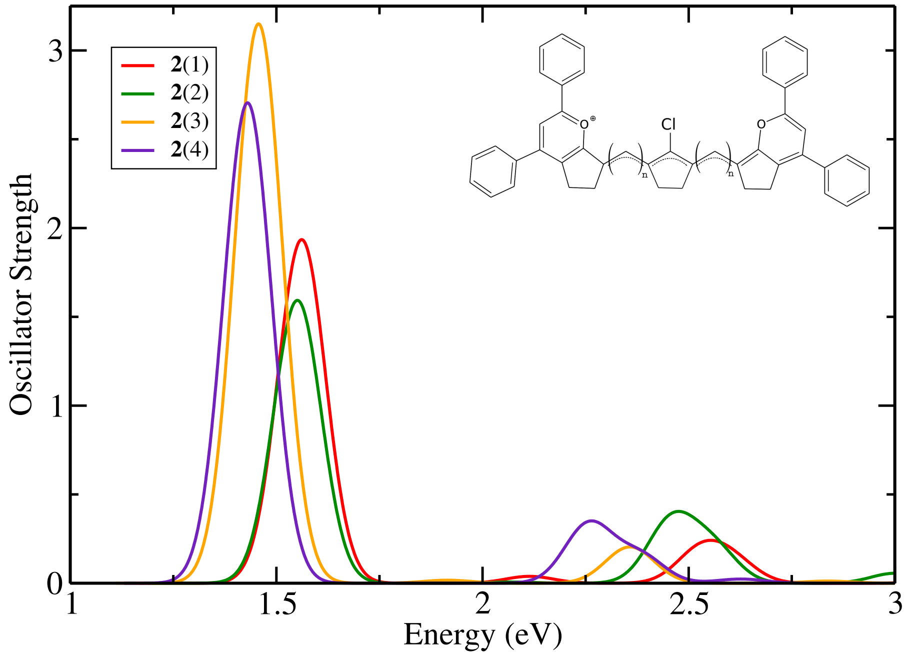

Figure S6: Theoretical absorbance spectra for molecules **2**(n) with n=1 to 4 in solvent ODCB at TD-DFT/B3LYP/TZP level with 5 excited states. The inset shows the basic structure for the molecule.

Table S5: Vertical excitation transitions S0-S1 for molecules **2**(n) with n=1 to 4 in solvent ODCB at TD-DFT/B3LYP/TZP.

	E_{exc} (eV)	f_{osc}
**2(1)**	1.56	1.93
**2(2)**	1.55	1.59
**2(3)**	1.45	3.15
**2(4)**	1.43	2.70

Table S6: Vertical excitation transitions (eV), main orbitals electronic transitions and oscillator strength (f_{osc}) for molecules **2**(n) with n=1 to 4 in solvent ODCB at TD-DFT/B3LYP/TZP level. The main orbitals involved in the first excitation of each molecule are its HOMO and LUMO.

	State	Transition (>10%)	E_{exc} (eV)	Osc.Stren.
**2(1)**	1	**175-176 (96%)**	1.56	1.93
	2	175-177 (85%)<br>174-176 (13%)	2.11	0.04
	3	175-178 (94%)	2.53	0.18
	4	174-176 (80%)<br>175-177 (10%)	2.61	0.12
	5	175-179 (70%)<br>172-176 (20%)	3.14	0.40
**2(2)**	1	**182-183 (96%)**	1.55	1.59
	2	182-184 (70%)<br>181-183 (29%)	2.06	0.00
	3	181-183 (65%)<br>182-184 (24%)	2.46	0.35
	4	182-185 (92%)	2.56	0.20
	5	182-186 (79%)	3.00	0.05
**2(3)**	1	**189-190 (96%)**	1.46	3.15
	2	189-191 (70%)<br>188-190 (29%)	1.91	0.02
	3	189-192 (91%)	2.35	0.13
	4	188-190 (61%)<br>189-191 (23%)	2.37	0.07
	5	187-190 (60%)<br>188-191 (38%)	2.84	0.01

**2(4)**	1	**196-197 (96%)**	1.43	2.70
	2	196-198 (57%)<br>195-197 (43%)	1.84	0.00
	3	195-197 (50%)<br>196-198 (36%)	2.26	0.33
	4	196-199 (89%)	2.38	0.16
	5	195-198 (66%)<br>194-197 (30%)	2.63	0.02

6 Conformation analysis

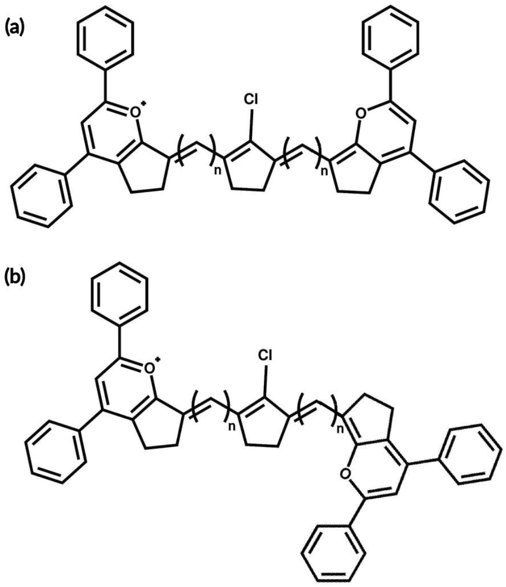

Figure S7: (a) trans-trans and (b) trans-cis structure for molecules **2**(n).

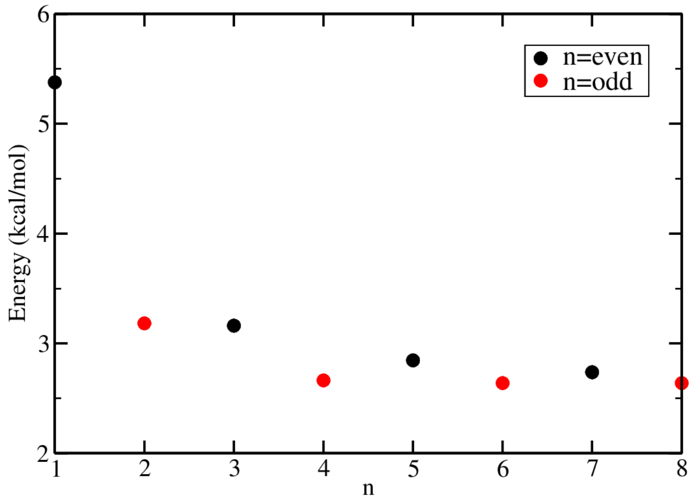

Figure S8: Energy difference between different conformers. For the first group (n=odd), trans-trans conformer is more stable. For the second group (n=even), trans-cis conformer is more stable.

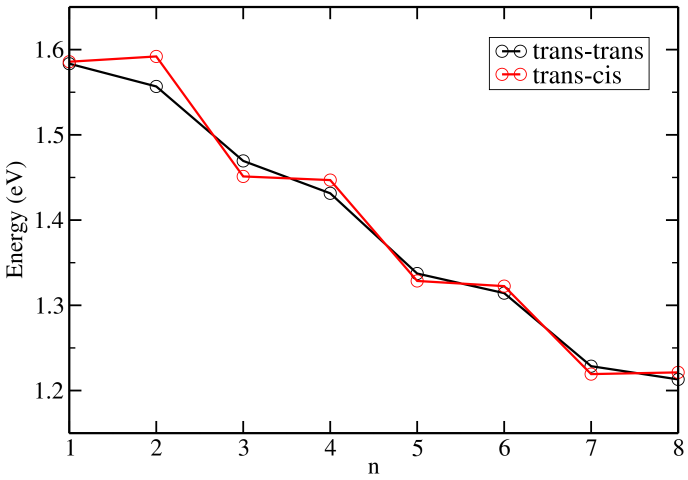

Figure S9: Main absorption peak for all trans-trans and trans-cis conformers

Table S7: Vertical excitation transitions (eV), main orbitals electronic transitions and oscillator strength (f_{osc}) for molecules **2**(n) trans-cis, with n=1 to 8 in gas phase at TD-DFT/B3LYP/TZP level. The main orbitals involved in the first excitation of each molecule are its HOMO and LUMO.

		**trans-cis**		
	**State**	**Transition (>10%)**	E_{exc} **(eV)**	f_{osc}
**2(1)**	1	**175-176 (97%)**	1.59	1.72
	2	175-177 (82%)<br>174-176 (16%)	2.18	0.01
	3	175-178 (79%)<br>174-176 (11%)	2.56	0.09
	4	174-176 (66%)<br>175-178 (14%)<br>175-177 (10%)	2.60	0.10
	5	173-176 (86%)	3.10	0.16
**2(2)**	1	**182-183 (96%)**	1.59	2.23
	2	182-184 (71%)<br>181-183 (28%)	2.10	0.01

	3	181-183 (66%)<br>182-184 (23%)	2.48	0.05
	4	182-185 (92%)	2.58	0.10
	5	181-184 (53%)<br>180-183 (44%)	3.01	0.00
**2(3)**	1	**189-190 (96%)**	1.45	2.62
	2	189-191 (69%)<br>188-190 (30%)	1.92	0.00
	3	189-192 (45%)<br>188-190 (31%)<br>189-191 (14%)	2.35	0.02
	4	189-192 (46%)<br>188-190 (30%)	2.36	0.25
	5	187-190 (62%)<br>188-191 (35%)	2.84	0.01
**2(4)**	1	**196-197 (96%)**	1.45	3.31
	2	196-198 (57%)<br>195-197 (43%)	1.85	0.00
	3	195-197 (49%)<br>196-198 (35%)<br>196-200 (11%)	2.28	0.04
	4	196-199 (89%)	2.37	0.16
	5	195-198 (64%)<br>194-197 (32%)	2.65	0.02
**2(5)**	1	**203-204 (96%)**	1.33	3.70
	2	203-205 (57%)<br>202-204 (42%)	1.72	0.00

	3	203-206 (50%)<br>202-204 (20%)<br>203-205 (16%)	2.18	0.00
	4	203-206 (37%)<br>202-204 (28%)<br>203-205 (18%)	2.19	0.20
	5	201-204 (56%)<br>202-205 (41%)	2.50	0.01
**2(6)**	1	**210-211 (96%)**	1.32	4.37
	2	209-211 (51%)<br>210-212 (48%)	1.65	0.00
	3	210-212 (43%)<br>209-211 (41%)<br>210-214 (11%)	2.13	0.03
	4	210-213 (85%)	2.20	0.08
	5	209-212 (58%)<br>208-211 (39%)	2.35	0.00
**2(7)**	1	**217-218 (96%)**	1.22	4.70
	2	217-219 (50%)<br>216-218 (50%)	1.54	0.00
	3	217-220 (75%)<br>215-218 (13%)	2.03	0.00
	4	216-218 (38%)<br>217-219 (37%)<br>217-221 (13%)	2.06	0.05
	5	215-218 (52%)<br>216-219 (44%)	2.21	0.01

**2(8)**	1	**224-225 (96%)**	1.22	4.915.38
	2	223-225 (55%)<br>224-226 (44%)	1.50	0.00
	3	224-226 (47%)<br>223-225 (36%)	2.01	0.02
	4	224-227 (77%)<br>223-226 (12%)	2.04	0.03
	5	223-226 (51%)<br>222-225 (46%)	2.10	0.00

7 Benchmark

7.1 ωB97X/TZP

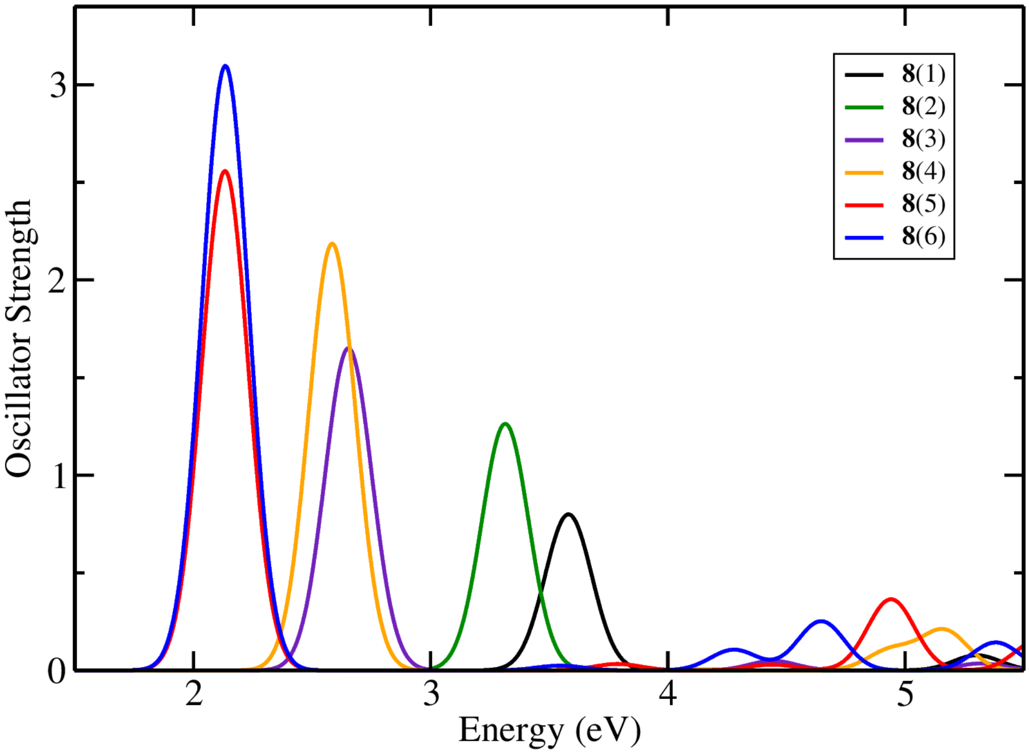

Figure S10: Theoretical absorbance spectra for molecules **8**(n) with n=1 to 6 in gas-phase at TD-DFT/ω97BX/TZP level with 5 excited states.

Table S8: Vertical excitation transitions (eV), main orbitals electronic transitions and oscillator strength (f_{osc}) for molecules **8**(n) with n=1 to 6 in gas phase at TD-DFT/ω97BX/TZP level. The main orbitals involved in the first excitation of each molecule are its HOMO and LUMO.

	**State**	**Transition (>10%)**	E_{exc} **(eV)**	f_{osc}
**8(1)**	1	**41-42 (97%)**	3.58	0.80
	2	40-42 (95%)	5.30	0.08
	3	39-42 (97%)	5.73	0.00
	4	41-43 (52%)<br>41-46 (27%)<br>41-48 (11%)	6.55	0.00

	5	41-44 (87%)	6.72	0.01
**8(2)**	1	**48-49 (96%)**	3.31	1.26
	2	47-49 (85%)‘	5.16	0.00
	3	46-49 (94%)	5.79	0.16
	4	45-49 (93%)	5.93	0.00
	5	48-50 (76%)	5.97	0.12
**8(3)**	1	**55-56 (96%)**	2.65	1.65
	2	54-56 (90%)	4.44	0.05
	3	55-57 (93%)	5.32	0.04
	4	51-56 (94%)	5.44	0.00
	5	53-56 (78%)	5.87	0.33
**8(4)**	1	**62-63 (93%)**	2.58	2.19
	2	61-63 (84%)	4.17	0.00
	3	62-64 (85%)	4.94	0.10
	4	60-63 (88%)	5.17	0.20
	5	62-68 (41%)<br>61-64 (38%)	6.17	0.14
**8(5)**	1	**69-70 (93%)**	2.13	2.56
	2	68-70 (88%)	3.79	0.03
	3	69-71 (89%)	4.44	0.03
	4	67-70 (80%)	4.94	0.36
	5	69-72 (72%)<br>68-71 (11%)	5.56	0.15
**8(6)**	1	**76-77 (90%)**	2.13	3.09
	2	75-77 (81%)<br>76-78 (11%)	3.54	0.02

	3	76-78 (81%)<br>75-77 (10%)	4.28	0.11
	4	74-77 (78%)<br>75-78 (11%)	4.65	0.25
	5	75-78 (40%)<br>76-79 (37%)	5.38	0.14

7.2 CAM-B3LYP/TZP

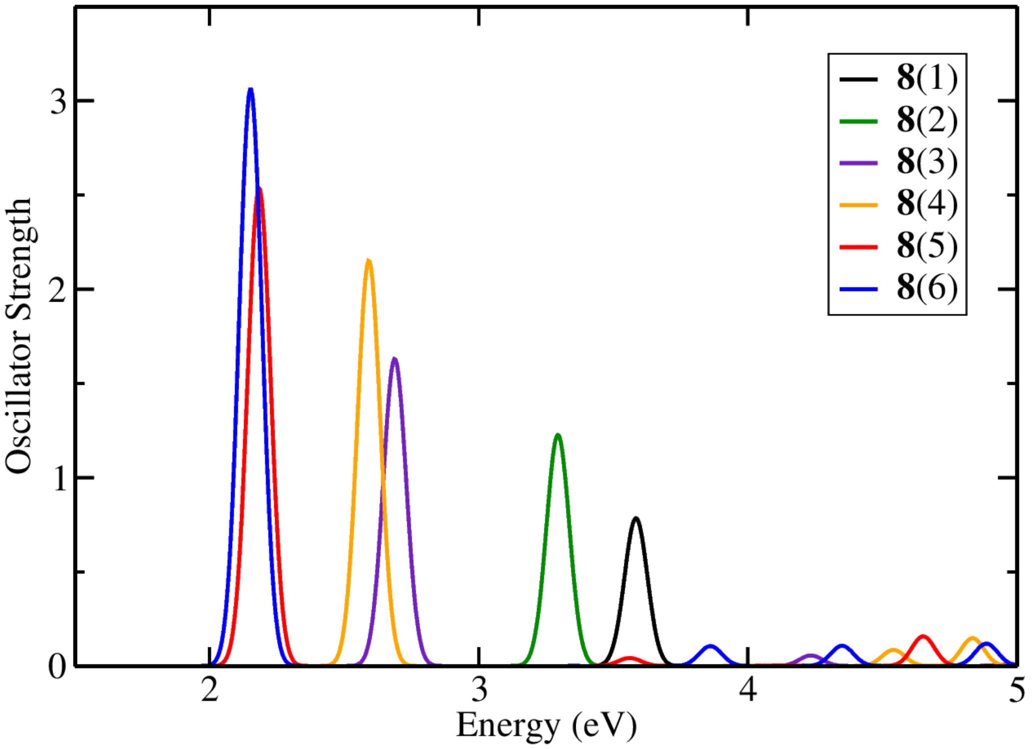

Figure S11: Theoretical absorbance spectra for molecules **8**(n) with n=1 to 6 in gas-phase at TD-DFT/CAM-B3LYP/TZP

Table S9: Vertical excitation transitions (eV), main orbitals electronic transitions and oscillator strength (f_{osc}) for molecules **8**(n) with n=1 to 6 in gas phase at TD-DFT/CAM-B3LYP/TZP level. The main orbitals involved in the first excitation of each molecule are its HOMO and LUMO.

	**State**	**Transition (>10%)**	E_{exc} **(eV)**	f_{osc}
**8(1)**	1	**41-42 (97%)**	3.58	0.78
	2	40-42 (97%)	5.09	0.08
	3	39-42 (98%)	5.50	0.00
	4	41-43 (90%)	5.97	0.00
	5	41-44 (93%)	6.04	0.01
**8(2)**	1	**48-49 (97%)**	3.30	1.23
	2	47-49 (93%)	4.99	0.00
	3	46-49 (98%)	5.43	0.16

	4	45-49 (97%)	5.53	0.00
	5	48-50 (98%)	5.56	0.09
**8(3)**	1	**55-56 (97%)**	2.69	1.63
	2	54-56 (95%)	4.23	0.06
	3	55-57 (95%)	4.97	0.01
	4	55-58 (65%)	5.52	0.00
	5	55-59 (75%)	6.58	0.00
**8(4)**	1	**62-63 (96%)**	2.59	2.15
	2	61-63 (96%)	3.98	0.00
	3	62-64 (97%)	4.59	0.09
	4	60-63 (95%)	4.83	0.15
	5	62-65 (69%)<br>62-67 (15%)	5.52	0.00
**8(5)**	1	**69-70 (96%)**	2.18	2.54
	2	68-70 (92%)	3.56	0.04
	3	69-71 (93%)	4.12	0.00
	4	67-70 (95%)	4.65	0.16
	5	69-70 (93%)	5.18	0.28
**8(6)**	1	**76-77 (94%)**	2.15	3.06
	2	75-77 (95%)	3.35	0.00
	3	76-78 (96%)	3.86	0.10
	4	74-77 (94%)	4.35	0.11
	5	75-78 (89%)	4.89	0.12

7.3 B2PLYP/def2-TZVP/def2-TZVP/C

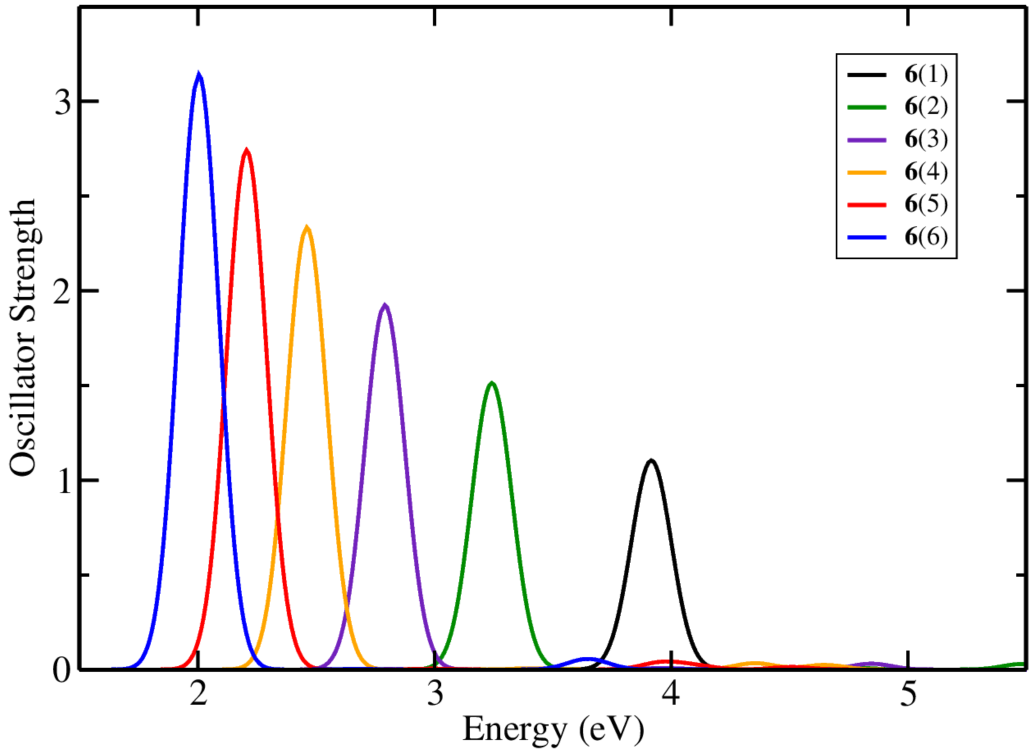

Figure S12: Theoretical absorbance spectra for molecules **6**(n) with n=1 to 6 in gas-phase at TD-DFT/B2PLYP/def2-TZVP/def2-TZVP/C

Table S10: Vertical excitation transitions (eV), main orbitals electronic transitions and oscillator strength (f_{osc}) for molecules **6**(n) with n=1 to 6 in gas phase at TD-DFT/B2PLYP/def2-TZVP/def2-TZVP/C level. The main orbitals involved in the first excitation of each molecule are its HOMO and LUMO.

	**State**	**Transition (>10%)**	E_{exc} **(eV)**	f_{osc}
**6(1)**	1	**25-26 (98%)**	3.92	1.10
	2	24-26 (97%)	6.04	0.01
	3	25-30 (95%)	6.62	0.00
	4	25-27 (97%)	6.24	0.03
	5	25-28 (92%)	6.64	0.00
**6(2)**	1	**32-33 (97%)**	3.24	1.51
	2	31-33 (93%)	4.76	0.01
	3	32-34 (92%)	5.48	0.03

	4	32-37 (94%)	6.25	0.00
	5	32-35 (89%)	6.23	0.00
**6(3)**	1	**39-40 (97%)**	2.79	1.92
	2	38-40 (90%)	3.97	0.01
	3	39-41 (89%)	4.85	0.03
	4	39-44 (92%)	6.02	0.00
	5	39-42 (79%)	5.97	0.00
**6(4)**	1	**46-47 (96%)**	2.46	2.33
	2	45-47 (88%)	3.41	0.00
	3	46-48 (87%)	4.35	0.03
	4	44-47 (94%)	4.65	0.02
	5	46-52 (89%)	5.86	0.00
**6(5)**	1	**53-54 (96%)**	2.21	2.74
	2	52-54 (87%)	2.99	0.00
	3	53-55 (86%)	3.96	0.04
	4	51-54 (93%)	4.10	0.02
	5	53-56 (56%)<br>52-55 (35%)	4.51	0.01
**6(6)**	1	**60-61 (95%)**	2.00	3.14
	2	59-61 (86%)	2.68	0.00
	3	60-62 (86%)	3.64	0.04
	4	58-61 (92%)	3.67	0.01
	5	59-62 (69%)<br>60-63 (22%)	4.05	0.00

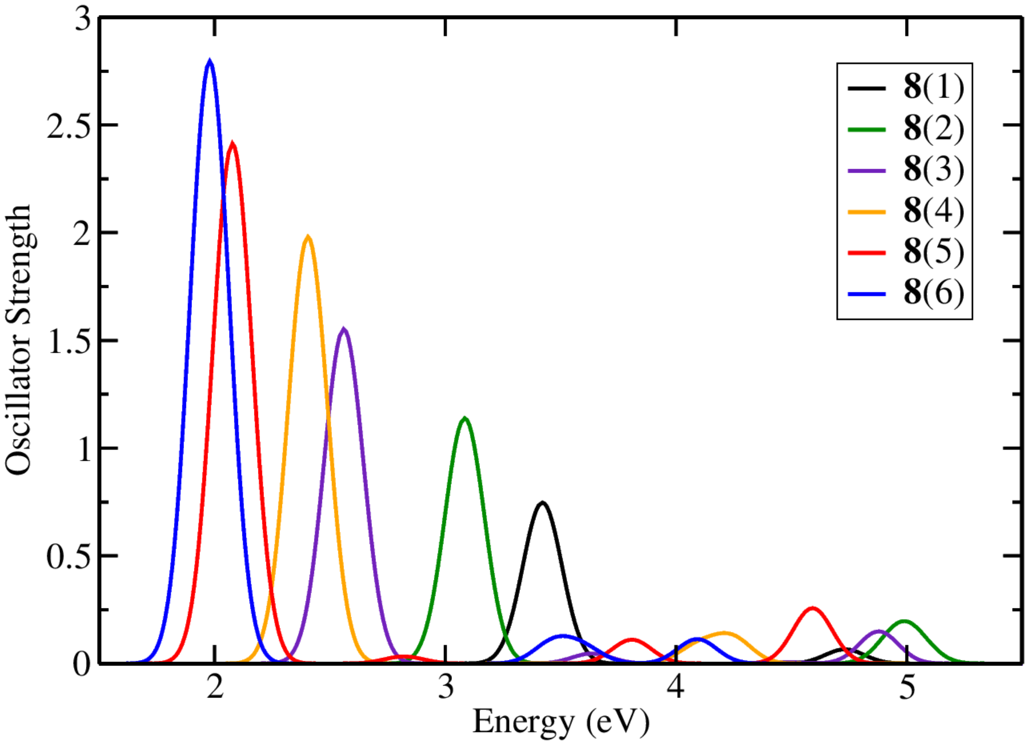

Figure S13: Theoretical absorbance spectra for molecules **8**(n) with n=1 to 6 in gas-phase at TD-DFT/B2PLYP/def2-TZVP/def2-TZVP/C

Table S11: Vertical excitation transitions (eV), main orbitals electronic transitions and oscillator strength (f_{osc}) for molecules **8**(n) with n=1 to 6 in gas phase at TD-DFT/B2PLYP/def2-TZVP/def2-TZVP/C level. The main orbitals involved in the first excitation of each molecule are its HOMO and LUMO.

	State	Transition (>10%)	E_{exc} (eV)	f_{osc}
**8(1)**	1	**40-41 (98%)**	3.42	0.75
	2	39-41 (98%)	4.73	0.7
	3	38-41 (97%)	5.36	0.09
	4	40-42 (94%)	6.12	0.00
	5	40-43 (90%)	6.13	0.00
**8(2)**	1	**47-48 (97%)**	3.08	1.14
	2	46-48 (97%)	4.48	0.01
	3	47-49 (98%)	5.04	0.08
	4	45-48 (97%)	4.96	0.13

	5	44-48 (94%)	5.16	0.00
**8(3)**	1	**54-55 (97%)**	2.56	1.55
	2	53-55 (95%)	3.63	0.05
	3	54-56 (95%)	4.47	0.01
	4	50-55 (96%)	4.98	0.00
	5	52-55 (97%)	4.88	0.15
**8(4)**	1	**61-62 (96%)**	2.40	1.98
	2	60-62 (95%)	3.34	0.00
	3	61-63 (93%)	4.09	0.08
	4	59-62 (96%)	4.24	0.11
	5	58-62 (94%)	4.92	0.00
**8(5)**	1	**68-69 (96%)**	2.08	2.41
	2	67-69 (89%)	2.82	0.03
	3	68-70 (88%)	3.76	0.00
	4	66-69 (95%)	3.81	0.11
	5	68-71 (93%)	4.59	0.26
**8(6)**	1	**75-76 (95%)**	1.98	2.79
	2	74-76 (93%)	2.67	0.00
	3	75-77 (93%)	3.47	0.10
	4	73-76 (95%)	3.60	0.07
	5	74-77 (91%)	4.09	0.11

7.4 B3LYP/AUG/ATZP

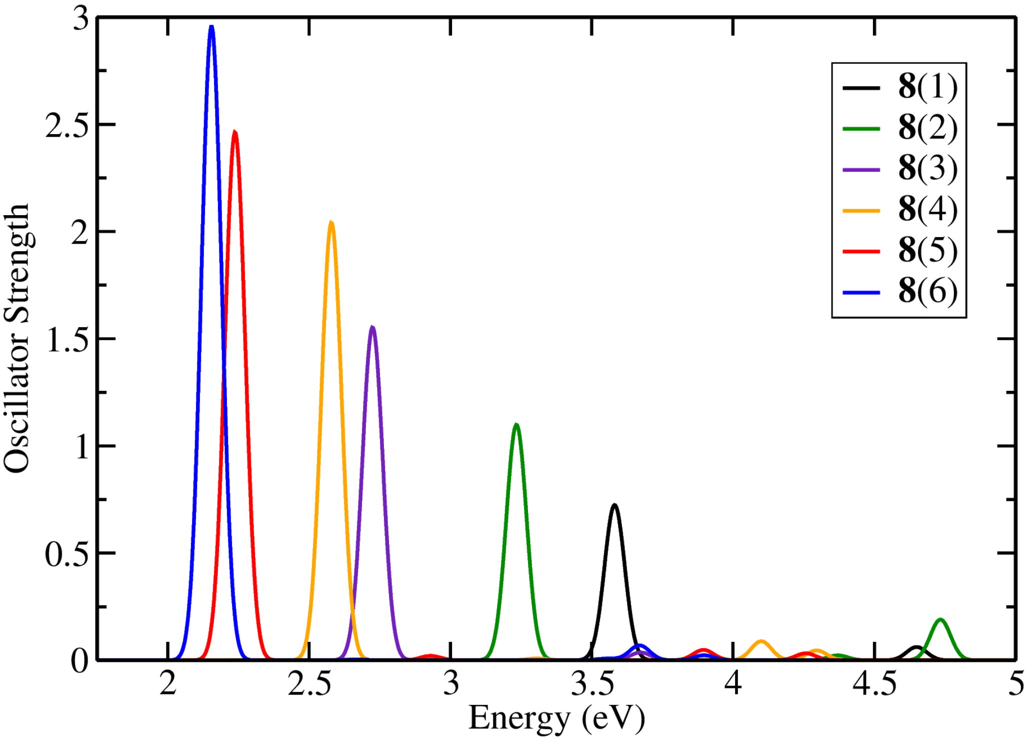

Figure S14: Theoretical absorbance spectra for molecules **8**(n) with n=1 to 6 in gas-phase at TD-DFT/B3LYP/AUG/ATZP

Table S12: Vertical excitation transitions (eV), main orbitals electronic transitions and oscillator strength (f_{osc}) for molecules **8**(n) with n=1 to 6 in gas phase at TD-DFT/B3LYP/AUG/ATZP level. The main orbitals involved in the first excitation of each molecule are its HOMO and LUMO.

	**State**	**Transition (>10%)**	E_{exc} **(eV)**	f_{osc}
**8(1)**	1	**41-42 (98%)**	3.58	0.72
	2	40-42 (96%)	4.65	0.06
	3	39-42 (100%)	5.01	0.00
	4	41-42 (98%)	5.36	0.00
	5	41-44 (99%)	5.42	0.01
**8(2)**	1	**48-49 (98%)**	3.23	1.1
	2	47-49 (78%)<br>48-50 (21%)	4.37	0.02

	3	46-49 (97%)	4.73	0.19
	4	45-49 (100%)	4.78	0.00
	5	48-50 (76%)<br>47-49 (19%)	5.17	0.04
**8(3)**	1	**55-56 (99%)**	2.72	1.55
	2	54-56 (81%)<br>55-57 (18%)	3.67	0.04
	3	55-57 (67%)<br>52-56 (19%)<br>54-56 (11%)	5.54	0.00
	4	51-56 (99%)	4.63	0.00
	5	55-58 (97%)	4.76	0.00
**8(4)**	1	**62-63 (99%)**	2.58	2.04
	2	61-63 (72%)<br>62-64 (28%)	3.30	0.01
	3	60-63 (94%)	4.10	0.01
	4	62-64 (70%)	4.29	0.05
	5	59-63 (99%)	4.56	0.00
**8(5)**	1	**69-70 (99%)**	2.24	2.46
	2	68-70 (69%)	2.93	0.02
	3	67-70 (85%)	2.89	0.04
	4	69-71 (63%)<br>68-70 (25%)	3.90	0.00
	5	69-72 (55%)<br>68-71 (43%)	4.25	0.03

**8(6)**	1	**76-77 (99%)**	2.15	2.96
	2	75-77 (68%)<br>76-78 (31%)	2.67	0.00
	3	74-77 (82%)<br>75-78 (15%)	3.55	0.01
	4	76-78 (67%)<br>75-77 (30%)	3.67	0.07
	5	75-78 (56%)<br>76-79 (36%)	3.90	0.02

7.5 ωB97X/AUG/ATZP

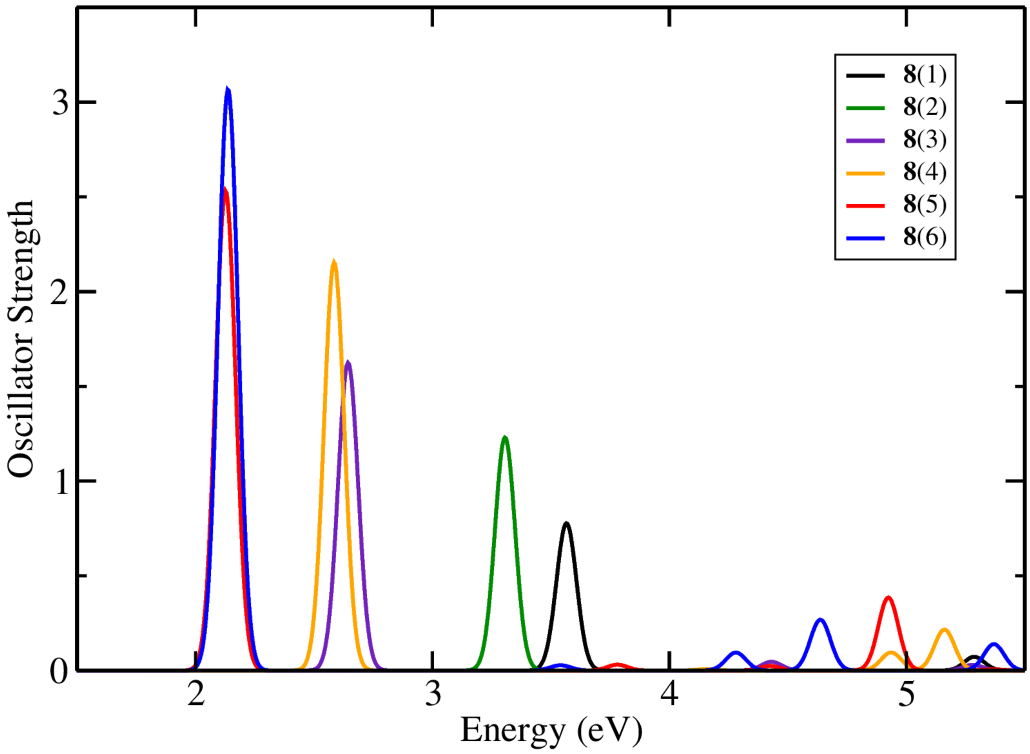

Figure S15: Theoretical absorbance spectra for molecules **8**(n) with n=1 to 6 in gas-phase at TD-DFT/ωB97X/AUG/ATZP

Table S13: Vertical excitation transitions (eV), main orbitals electronic transitions and oscillator strength (f_{osc}) for molecules **8**(n) with n=1 to 6 in gas phase at TD-DFT/ωB97X/AUG/ATZP level. The main orbitals involved in the first excitation of each molecule are its HOMO and LUMO.

	**State**	**Transition (>10%)**	E_{exc} **(eV)**	f_{osc}
**8(1)**	1	**41-42 (97%)**	3.57	0.78
	2	40-42 (95%)	5.29	0.07
	3	39-42 (97%)	5.73	0.00
	4	41-43 (70%)	6.33	0.00
	5	41-44 (75%)	6.58	0.01
**8(2)**	1	**48-49 (95%)**	3.30	1.23
	2	47-49 (83%)<br>48-50 (11%)	5.14	0.00
	3	46-49 (93%)	5.80	0.16
	4	48-50 (84%)<br>47-49 (11%)	5.93	0.11
	5	45-49 (92%)	5.95	0.00
**8(3)**	1	**55-56 (95%)**	2.64	1.62
	2	54-56 (90%)	4.43	0.05
	3	55-57 (92%)	5.28	0.03
	4	55-58 (61%)<br>55-61 (15%)	5.80	0.00
	5	55-59 (68%)	6.06	0.00
**8(4)**	1	**62-63 (93%)**	2.58	2.15
	2	61-63 (83%)	4.16	0.01
	3	62-64 (84%)	4.94	0.09
	4	60-63 (86%)	5.16	0.22
	5	62-65 (64%)	5.82	0.00

**8(5)**	1	**69-70 (93%)**	2.13	2.53
	2	68-70 (87%)	3.78	0.03
	3	69-71 (89%)	4.42	0.02
	4	67-70 (78%)	4.92	0.38
	5	66-70 (82%)	5.37	0.01
**8(6)**	1	**76-77 (90%)**	2.13	3.07
	2	75-77 (80%)<br>76-78 (12%)	3.54	0.03
	3	76-78 (80%)<br>75-77 (11%)	4.28	0.09
	4	74-77 (76%)<br>75-78 (12%)	4.64	0.27
	5	76-82 (39%)<br>75-78 (37%)<br>74-77 (11%)	5.37	0.14

8 Vertical TD-DFT excitation and main orbitals electronic transitions for molecules 1-8(n) in gas phase at B3LYP/TZP level

Table S14: Vertical excitation transitions (eV), main orbitals electronic transitions and oscillator strength (f_{osc}) for molecules1 1-8(n) in gas phase at TD-DFT/B3LYP/TZP level. The main orbitals involved in the first excitation of each molecule are its HOMO and LUMO.

	State	Transition (>10%)	E_{exc} (eV)	f_{osc}
**1(3)**	1	**201-202 (96%)**	1.46	3.17
	2	201-203 (58%)<br>200-202 (41%)	1.93	0.01
	3	200-202 (52%)<br>201-203 (35%)	2.33	0.02
	4	201-204 (90%)	2.39	0.06
	5	199-202 (67%)<br>200-203 (30%)	2.79	0.00
**2(1)**	1	**175-176 (96%)**	1.58	1.96
	2	175-177 (85%)<br>174-176 (13%)	2.15	0.04
	3	175-178 (93%)	2.56	0.17
	4	174-176 (81%)<br>175-177 (10%)	2.62	0.13
	5	173-176 (92%)	3.10	0.86
**2(2)**	1	**182-183 (96%)**	1.55	1.61
	2	182-184 (71%)<br>181-183 (29%)	2.08	0.00

	3	181-183 (65%)<br>182-184 (24%)	2.47	0.34
	4	182-185 (92%)	2.56	0.19
	5	182-186 (53%)<br>181-184 (17%)<br>180-183 (14%)	3.00	0.45
**2(3)**	1	**189-190 (96%)**	1.47	3.16
	2	189-191 (70%)<br>188-190 (29%)	1.93	0.01
	3	189-192 (91%)	2.36	0.13
	4	188-190 (61%)<br>189-191 (23%)	2.38	0.07
	5	187-190 (62%)<br>188-191 (35%)	2.84	0.00
**2(4)**	1	**196-197 (96%)**	1.43	2.73
	2	196-198 (57%)<br>195-197 (43%)	1.84	0.00
	3	195-197 (50%)<br>196-198 (35%)<br>196-200 (11%)	2.26	0.32
	4	196-199 (89%)	2.37	0.16
	5	195-198 (65%)<br>194-197 (32%)	2.64	0.02
**2(5)**	1	**203-204 (96%)**	1.34	4.26
	2	203-205 (58%)<br>202-204 (42%)	1.72	0.01
	3	203-206 (88%)	2.18	0.06

	4	202-204 (48%)<br>203-205 (33%)<br>203-207 (15%)	2.20	0.06
	5	201-204 (56%)<br>202-205 (41%)	2.50	0.01
**2(6)**	1	**210-211 (96%)**	1.31	3.82
	2	209-211 (51%)<br>210-212 (48%)	1.64	0.00
	3	210-212 (43%)<br>209-211 (41%)<br>210-214 (10%)	2.11	0.28
	4	210-213 (84%)	2.20	0.07
	5	209-212 (58%)<br>208-211 (39%)	2.34	0.00
**2(7)**	1	**217-218 (96%)**	1.23	5.30
	2	217-219 (50%)<br>216-218 (49%)	1.55	0.00
	3	217-220 (83%)<br>215-218 (12%)	2.02	0.01
	4	216-218 (41%)<br>217-219 (40%)<br>217-221 (14%)	2.07	0.05
	5	215-218 (52%)<br>216-219 (43%)	2.22	0.01
**2(8)**	1	**224-225 (96%)**	1.21	4.91
	2	223-225 (56%)<br>224-226 (43%)	1.48	0.00

	3	224-226 (48%)<br>223-225 (37%)	1.99	0.23
	4	224-227 (78%)<br>223-226 (12%)	2.05	0.02
	5	223-226 (52%)<br>222-225 (45%)	2.09	0.00
**3(1)**	1	**167-168 (96%)**	1.59	1.98
	2	167-169 (88%)<br>166-168 (11%)	2.14	0.04
	3	167-170 (93%)	2.55	0.21
	4	166-168 (81%)	2.70	0.16
	5	165-168 (54%)<br>167-171 (23%)<br>164-168 (15%)	3.15	0.10
**3(2)**	1	174-175 (96%)	1.58	1.61
	2	174-176 (64%)<br>173-175 (35%)	2.10	0.00
	3	173-175 (58%)<br>174-176 (29%)<br>174-178 (11%)	2.48	0.38
	4	174-177 (93%)	2.57	0.22
	5	173-176 (72%)<br>172-175 (18%)	3.03	0.02
**3(3)**	1	**181-182 (96%)**	1.47	3.19
	2	181-183 (74%)<br>180-182 (25%)	1.92	0.02
	3	181-184 (92%)	2.34	0.16

	4	180-182 (64%)<br>181-183 (18%)<br>181-185 (14%)	2.40	0.09
	5	179-182 (67%)<br>180-183 (31%)	2.87	0.00
**3(4)**	1	**188-189 (96%)**	1.45	2.75
	2	188-190 (52%)<br>187-189 (48%)	1.87	0.00
	3	187-189 (44%)<br>188-190 (40%)<br>188-192 (12%)	2.28	0.36
	4	188-191 (90%)	2.39	0.16
	5	187-190 (72%)<br>186-189 (24%)	2.66	0.02
**4(1)**	1	**135-136 (98%)**	1.71	2.08
	2	134-136 (58%)<br>135-137 (41%)	2.31	0.00
	3	135-137 (51%)<br>134-136 (35%)	2.72	0.07
	4	133-136 (78%)<br>131-136 (12%)	2.95	0.03
	5	131-136 (66%)<br>135-138 (23%)	3.10	0.07
**4(2)**	1	**142-143 (98%)**	1.68	2.86
	2	141-143 (57%)<br>142-144 (43%)	2.17	0.00

	3	142-144 (51%)<br>141-143 (37%)	2.69	0.02
	4	140-143 (81%)	2.92	0.01
	5	141-144 (62%)<br>142-145 (34%)	3.07	0.00
**4(3)**	1	**149-150 (97%)**	1.53	3.17
	2	148-150 (55%)<br>149-151 (45%)	1.99	0.00
	3	149-151 (50%)<br>148-150 (40%)	2.53	0.06
	4	147-150 (68%)<br>149-152 (31%)	2.64	0.00
	5	148-151 (41%)<br>149-152 (39%)<br>147-150 (16%)	2.82	0.00
**4(4)**	1	**156-157 (98%)**	1.51	3.95
	2	155-157 (57%)<br>156-158 (42%)	1.89	0.00
	3	156-158 (53%)<br>155-157 (38%)	2.50	0.03
	4	154-157 (73%)<br>155-158 (17%)	2.62	0.00
	5	156-159 (49%)<br>155-158 (47%)	2.67	0.00
**5(1)**	1	**168-169 (96%)**	1.67	2.08
	2	168-170 (83%)<br>167-169 (16%)	2.24	0.03

	3	167-169 (79%)<br>168-170 (13%)	2.67	0.13
	4	168-171 (92%)	2.68	0.21
	5	166-169 (91%)	3.14	0.15
**5(2)**	1	**175-176 (96%)**	1.55	1.67
	2	175-177 (78%)<br>174-176 (21%)	2.09	0.02
	3	174-176 (69%)<br>175-177 (16%)<br>175-179 (10%)	2.53	0.30
	4	175-178 (90%)	2.55	0.20
	5	175-179 (75%)<br>172-176 (10%)	2.99	0.07
**5(3)**	1	**182-183 (96%)**	1.51	3.28
	2	182-184 (66%)<br>181-183 (33%)	1.98	0.01
	3	181-183 (58%)<br>182-184 (27%)<br>182-186 (12%)	2.40	0.07
	4	182-185 (92%)	2.43	0.14
	5	181-184 (50%)<br>180-183 (48%)	2.88	0.00
**5(4)**	1	**189-190 (96%)**	1.42	2.80
	2	189-191 (63%)<br>188-190 (37%)	1.85	0.00

	3	188-190 (54%)<br>189-191 (29%)<br>189-193 (13%)	2.29	0.30
	4	189-192 (89%)	2.35	0.16
	5	188-191 (54%)<br>187-190 (44%)	2.68	0.01
**6(1)**	1	**26-27 (99%)**	4.15	1.16
	2	25-27 (56%)<br>26-28 (43%)	5.85	0.02
	3	26-29 (96%)	5.89	0.00
	4	26-30 (99%)	5.94	0.00
	5	26-31 (96%)	5.98	0.00

**6(2)**	1	**33-34 (99%)**	3.47	1.59
	2	32-34 (63%)<br>33-35 (36%)	4.75	0.01
	3	33-36 (95%)	5.44	0.00
	4	33-37 (98%)	5.46	0.00
	5	33.38 (96%)	5.53	0.00
**6(3)**	1	**40-41 (99%)**	3.00	2.03
	2	39-41 (65%)<br>40-42 (35%)	4.00	0.01
	3	40-42 (64%)<br>39-41 (34%)	5.10	0.01
	4	40-44 (62%)<br>40-43 (34%)	5.14	0.00
	5	40-43 (63%)<br>40-44 (34%)	5.15	0.00
**6(4)**	1	**47-48 (99%)**	2.67	2.47
	2	46-48 (65%)<br>47-49 (35%)	3.47	0.00
	3	47-49 (64%)<br>46-48 (34%)	4.59	0.01
	4	45-48 (80%)<br>46-49 (13%)	4.69	0.00
	5	47-51 (73%)<br>47-52 (23%)	4.93	0.00
**6(5)**	1	**54-55 (99%)**	2.40	2.92
	2	53-55 (64%)<br>54-56 (35%)	3.06	0.00

	3	52-55 (78%)<br>53-56 (17%)	4.17	0.00
	4	54-56 (63%)<br>53-55 (34%)	4.18	0.02
	5	54-57 (53%)<br>53-56 (44%)	4.42	0.00
**6(6)**	1	**61-62 (99%)**	2.20	3.35
	2	60-62 (64%)<br>61-62 (36%)	2.75	0.00
	3	59-62 (76%)<br>60-63 (19%)	3.75	0.00
	4	61-63 (63%)<br>60-62 (35%)	3.85	0.02
	5	61-64 (52%)<br>60-63 (44%)	4.00	0.00
**7(1)**	1	**33.34 (98%)**	3.71	0.83
	2	33-36 (99%)	5.41	0.00
	3	33-35 (99%)	5.42	0.00
	4	32-34 (71%)<br>33-37 (27%)	5.65	0.03
	5	33-38 (99%)	5.98	0.00
**7(2)**	1	**40-41 (99%)**	3.43	1.35
	2	39-41 (84%)<br>40-42 (15%)	4.42	0.02
	3	40-43 (97%)	5.40	0.00
	4	40-44 (97%)	5.43	0.00

	5	40-42 (80%)<br>39-41 (13%)	5.45	0.00
**7(3)**	1	**47-48 (99%)**	2.79	1.68
	2	46-48 (61%)<br>47-49 (39%)	3.92	0.02
	3	47-50 (90%)	4.90	0.00
	4	47-51 (87%)	4.90	0.00
	5	45-48 (70%)<br>47-52 (14%)	4.91	0.06
**7(4)**	1	**54-55 (99%)**	2.67	2.22
	2	53-55 (76%)<br>54-56 (23%)	3.35	0.01
	3	54-56 (74%)<br>53-55 (22%)	4.46	0.01
	4	52-55 (66%)<br>53-56 (33%)	4.65	0.00
	5	54-59 (45%)<br>53-56 (38%)<br>52-55 (15%)	4.89	0.00
**7(5)**	1	**61-62 (99%)**	2.28	2.59
	2	60-62 (61%)<br>61-63 (38%)	3.02	0.01
	3	59-62 (85%)<br>61-64 (11%)	3.93	0.04
	4	61-63 (61%)<br>60-62 (37%)	4.08	0.00

	5	61-64 (55%)<br>60-63 (41%)	4.34	0.05
**7(6)**	1	**68-69 (99%)**	2.21	3.09
	2	67-69 (72%)<br>68-70 (28%)	2.70	0.00
	3	66-69 (59%)<br>67-70 (40%)	3.75	0.02
	4	68-70 (71%)<br>67-69 (27%)	3.78	0.03
	5	68-71 (47%)<br>67-70 (32%)<br>66-69 (20%)	3.99	0.00
**8(1)**	1	**41-42 (98%)**	3.60	0.74
	2	40-42 (96%)	4.66	0.06
	3	39-42 (100%)	5.01	0.00
	4	41-44 (96%)	5.52	0.00
	5	41-43 (99%)	5.53	0.00
**8(2)**	1	**48-49 (98%)**	3.25	1.11
	2	47-49 (79%)<br>48-50 (20%)	4.38	0.02
	3	46-49 (97%)	4.72	0.19
	4	45-49 (100%)	4.75	0.00
	5	48-50 (77%)<br>47-49 (18%)	5.24	0.04
**8(3)**	1	**55-56 (99%)**	2.74	1.57
	2	54-56 (81%)<br>55-57 (18%)	3.68	0.04

	3	55-57 (65%)<br>52-56 (23%)<br>54-56 (11%)	4.58	0.00
	4	51-56 (99%)	4.63	0.00
	5	53-56 (85%)<br>55-60 (12%)	4.87	0.08
**8(4)**	1	**62-53 (99%)**	2.56	2.06
	2	61-63 (72%)<br>62-64 (28%)	3.31	0.01
	3	60-63 (94%)	4.09	0.09
	4	62-64 (70%)<br>61-63 (26%)	4.33	0.04
	5	59-63 (100%)	4.54	0.00
**8(5)**	1	**69-70 (99%)**	2.25	2.47
	2	68-70 (69%)<br>69-70 (30%)	2.94	0.02
	3	67-70 (85%)	3.89	0.04
	4	69-71 (62%)<br>68-70 (25%)<br>66-70 (11%)	3.92	0.00
	5	69-72 (53%)<br>68-71 (45%)	4.27	0.03
**8(6)**	1	**76-77 (99%)**	2.16	2.97
	2	75-77 (68%)<br>76-78 (32%)	2.67	0.00
	3	74-77 (81%)<br>75-78 (16%)	3.55	0.00

	4	76-78 (67%)<br>75-77 (30%)	3.69	0.07
	5	75-78 (56%)<br>76-79 (36%)	3.90	0.02
